# Transcriptomic response of *Xanthomonas campestris* during xanthan gum production to glutamate concentration

**DOI:** 10.1038/s41598-026-43665-8

**Published:** 2026-03-13

**Authors:** Lu Wang, Xinmin Song, Chuanfu Ji, Maozhang Tian, Caiyun Xie, Min Gou, Wenfeng Song, Yueqin Tang

**Affiliations:** 1 Research Institute of Petroleum Exploration & Development, CNPC, Beijing, 100083 China; 2 State Key Laboratory of Enhanced Oil & Gas Recovery, Beijing, 100083 China; 3 College of Architecture and Environment, Sichuan University, No. 24 South Section 1 First Ring Road, Chengdu, 610065 China

**Keywords:** Xanthomonas, Xanthan gum, Glutamate concentration, Nitrogen limitation, Two-component system, Biopolymers, Transcriptomics

## Abstract

The molecular mechanisms underlying the influence of nitrogen sources on xanthan gum production remain poorly understood. This study compared the effects of two nitrogen sources (NH_4_Cl and glutamate) on xanthan gum production in *Xanthomonas campestris*. Additionally, the transcriptomic responses to different glutamate concentrations during fermentation were investigated. The findings revealed that glutamate, particularly at a lower concentration of 1 g/L, significantly enhanced xanthan gum yield and viscosity. Comparative transcriptomic analysis across different fermentation stages revealed that nitrogen limitation, observed on day 4 under 1 g/L glutamate and day 6 under 2 g/L glutamate, favored xanthan gum biosynthesis. Furthermore, low glutamate concentration improved xanthan gum production primarily by altering nitrogen assimilation and redirecting carbon metabolic fluxes through modulation in the expression of genes (*rpoN*, *rpfC*, and *rpfG*) associated within two-component regulatory system. These results may provide valuable guidance for xanthan gum production using genetically engineered bacteria.

**Supplementary Information:**

The online version contains supplementary material available at 10.1038/s41598-026-43665-8.

## Introduction

Xanthan gum, the most commercially significant microbial exopolysaccharides (EPS), has an average molecular weight of 2.0 × 10^6^ — 5.0 × 10^7^ Da. Its structure comprises repeating pentasaccharide units, each containing: (1) a cellulose-like backbone of *β*-1,4-linked D-glucose dimers, and (2) a trisaccharide side chain (mannose-glucuronic acid-mannose) attached to alternating glucose residues^1^. Acetate and pyruvate groups are non-stoichiometrically attached to the mannose residues^2^. Xanthan gum serves as a thickener, stabilizer, and emulsifier in diverse industries including food, petroleum recovery, pharmaceuticals, and cosmetics, etc.^3^. With global production reaching ~ 50,000 tons annually, the xanthan market is expected to attain USD 1.2 billion by 2030^4,5^. To meet steadily rising demand (growing at a rate of 5.6% annually since 2019) for xanthan gum, there is an urgent need to enhance production yields while optimizing core characteristics, such as viscosity and molecular weight.

Generally, xanthan gum is industrially produced through fermentation using various strains of the genus *Xanthomonas* (primarily *X*. *campestris*) via complex biochemical reactions. Over the years, researchers have mainly focused on optimizing media component, fermentation conditions, and microbial strains, significantly improving production yields^1,6–8^. However, further advancements in fermentation control or engineered strain development require deeper understanding of xanthan biosynthesis molecular mechanisms in *Xanthomonas*. Therefore, genome analyses of *Xanthomonas* species have been conducted, providing new insights into gene functions and metabolic pathway reconstruction for xanthan gum production^9–13^. In addition, comparative transcriptomics, a key approach for investigating molecular mechanism, has been widely applied to *Xanthomonas* species^14–16^. While these studies revealed cellular responses to environmental factors, they primarily examined xanthan’s role as a virulence factor in plant pathogenesis. This emphasis has created a significant knowledge gap regarding gene expression patterns during industrial xanthan production, with limited studies addressing this context. Alkhateeb et al. first reported that  *X campestris* pv. *campestris* B100 exhibits peak expression of xanthan precursors synthesis genes during the stationary phase, correlating with maximum gum production^17^. Schulte et al. demonstrated that methionine supplementation alters transcriptional profiles and identified the key regulator CysB^18^. Given these sparse but critical findings, expanding our understanding of cellular responses to diverse fermentation conditions is essential for enhancing xanthan gum production efficiency.

Nutrients composition, particularly carbon and nitrogen source, critically regulates xanthan gum production. These nutrients influence side-chain structure and xanthan polymer aggregation, leading to variations in molecular weight, viscosity, and yield of xanthan gum^19,20^. Conventionally, ammonium or nitrate salts serve as the primary nitrogen sources in both laboratory- and industrial-scale xanthan gum production. Several studies^21–23^ have demonstrated that amino acids like glutamate outperform other nitrogen sources (including ammonium) in enhancing xanthan gum yield. Proteomic analysis of *X. citri* confirms glutamate’s role in promoting cell growth, establishing its promise as a nitrogen source for xanthan biosynthesis^23^. However, xanthan yield exhibits concentration-dependent responses to glutamate, with excessive levels causing significant suppression of production^22^. Despite these empirical observations, the molecular mechanism through which glutamate concentration modulates xanthan production in *X. campestris* remains unexplored.

In this study, the effects of different concentrations of two nitrogen sources (NH_4_Cl and glutamate) on xanthan gum production in *X. campestris* were compared. Furthermore, the transcriptomic response of *X. campestris* to different glutamate concentrations during fermentation were investigated, which may provide the valuable guidance for enhancing the production of xanthan gum.

## Results and discussion

### Xanthan gum production

**Fig. 1:**
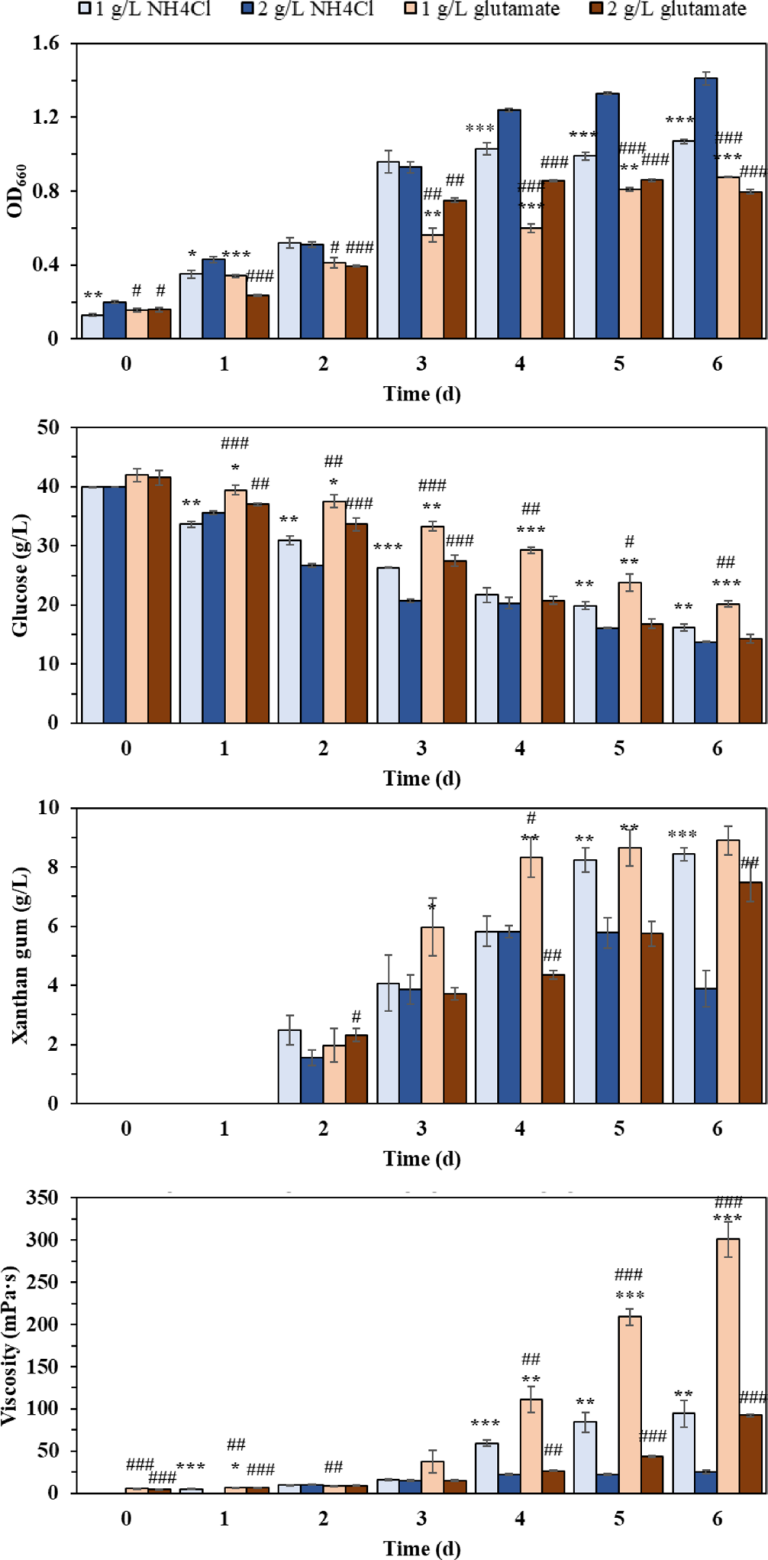
Changes in fermentation parameters under different nitrogen sources. Statistical significance was determined using an independent samples *t*-test. Significant differences between 2 g/L and 1 g/L are denoted as * (*p < 0.05, **p < 0.01, ***p < 0.001), while differences between NH_4_Cl and glutamate are marked with # (#*p* < 0.05, ##*p* < 0.01, ###*p* < 0.001).

**Fig. 2:**
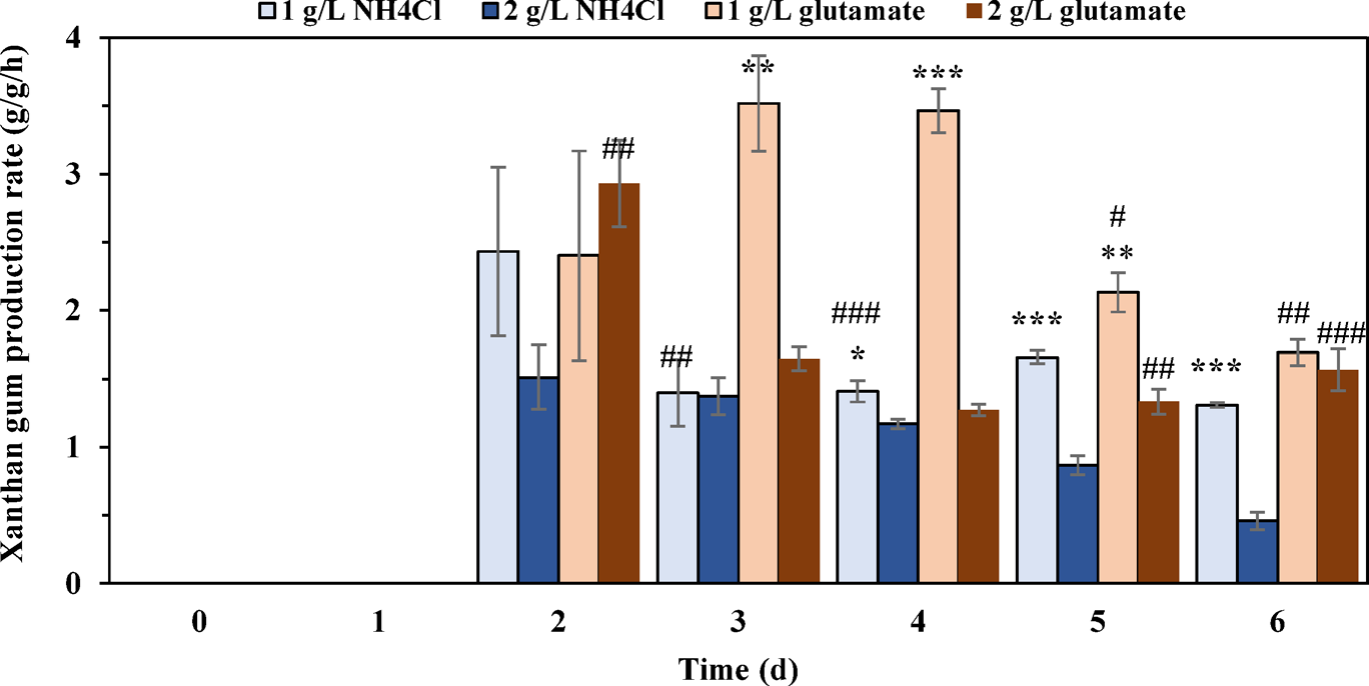
Xanthan gum production rate under different nitrogen sources. The significance analysis is the same as that in Fig. 1.

Figures 1 and 2 illustrated the effects of nitrogen source types (NH_4_Cl and glutamate) and concentrations (1 g/L and 2 g/L) on the production of xanthan gum. At 1 g/L NH₄Cl, cell growth entered the stationary phase after three days of fermentation. In contrast, with 2 g/L NH₄Cl, cells exhibited slow growth beyond day 3, accompanied by more pronounced glucose consumption (Fig. 1). Regarding xanthan gum synthesis, no xanthan gum was detected on day 1 under either concentration. With 1 g/L NH₄Cl, the xanthan gum yield increased markedly from days 1 to 5, stabilizing thereafter. With 2 g/L NH₄Cl, the yield reached a level comparable to the 1 g/L condition by day 4 but gradually declined afterwards. The viscosity of the fermentation broth also differed significantly: it gradually increased throughout fermentation at 1 g/L NH₄Cl, while remaining relatively constant at 2 g/L NH₄Cl. Both concentrations exhibited peak xanthan gum production rates on day 2 (2.43 g/g/h for 1 g/L; 1.52 g/g/h for 2 g/L), followed by gradual declines over time (Fig. 2).

When glutamate was used as the nitrogen source, a reduction in overall cell growth was observed compared to NH₄Cl (Fig. 1). At 1 g/L glutamate, cell growth increased gradually throughout fermentation, whereas at 2 g/L glutamate, the culture entered the stationary phase after three days. Notably, increasing the glutamate concentration from 1 g/L to 2 g/L markedly stimulated glucose consumption. Similar to the NH₄Cl results, higher glutamate concentration (2 g/L) decreased xanthan gum yield and broth viscosity. At 1 g/L glutamate, the xanthan gum yield increased from 1.97 g/L (day 2) to 8.32 g/L (day 4) and 8.91 g/L (day 6), with corresponding viscosities of 8.63, 110.97, and 301.00 mPa·s. In contrast, at 2 g/L glutamate, yields were 2.31 g/L (day 2), 4.36 g/L (day 4), and 7.48 g/L (day 6), while viscosities remained lower (9.50, 26.80, and 92.33 mPa·s, respectively). With 1 g/L glutamate, the xanthan gum production rate peaked on day 3 at 3.52 g/L/h before declining (Fig. 2). In contrast, with 2 g/L glutamate, the rate peaked earlier (day 2) before decreasing.

This study confirmed that nitrogen source concentration significantly influences both cell growth and xanthan gum accumulation. Compared to glutamate, NH₄Cl more effectively promoted cell growth, while both nitrogen sources demonstrated that lower concentrations were more beneficial for xanthan gum accumulation, with glutamate achieving the highest yield at 1 g/L. In xanthan gum production, both NH₄Cl and glutamate are commonly used nitrogen sources. Our flask experiments demonstrated that glutamate surpasses NH₄Cl in enhancing xanthan gum yield and viscosity, indicating its potential as a high-efficiency nitrogen source for subsequent medium optimization. However, its industrial-scale feasibility requires further evaluation. Previous studies using NH₄Cl or glutamate have established that high carbon-to-nitrogen ratios promote xanthan accumulation^24^. Our findings corroborate this conclusion, though the underlying mechanism remains unelucidated. To investigate how high C/N ratios enhance xanthan production, we examined the transcriptomic responses of *X. campestris* to varying glutamate concentrations.

### Transcriptomic analysis between different fermentation stages

**Fig. 3:**
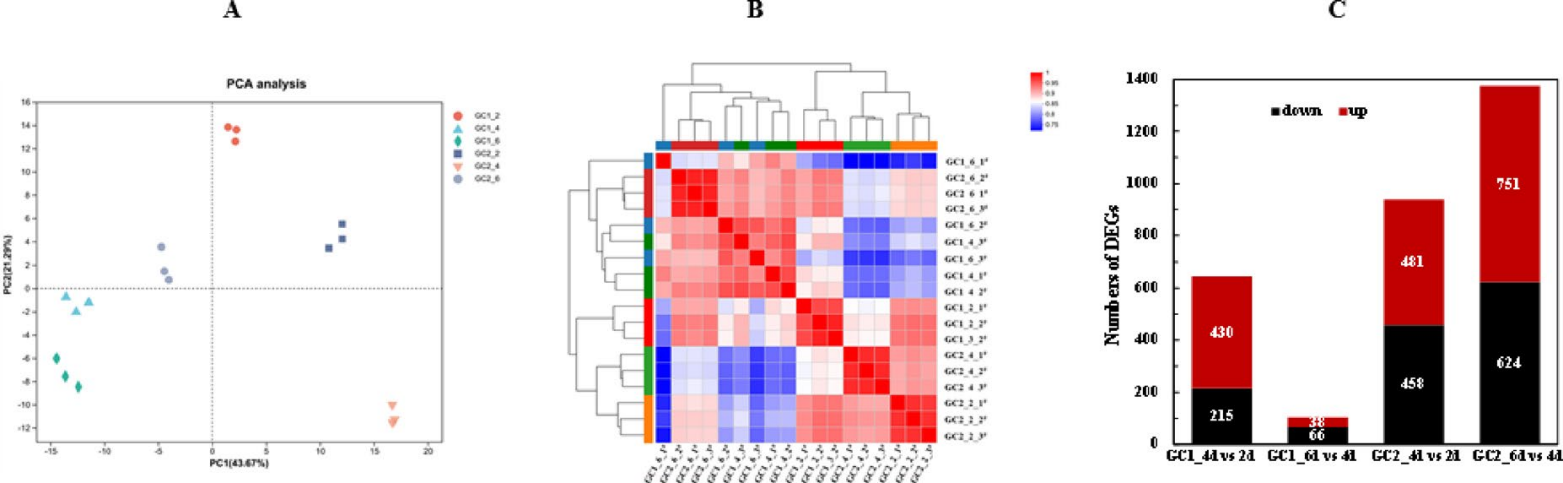
Principal component analysis (**A**) and heatmap of gene expression (**B**) of all transcriptome samples, and numbers of DEGs during different fermentation stages (**C**).

To investigate the response of strain E01 to different fermentation stages (days 2, 4, and 6), transcriptomic profiles from pairwise comparisons (GC1_4d vs. 2d, GC1_6d vs. 4d, GC2_4d vs. 2d, GC2_6d vs. 4d) were analyzed. Principal Component Analysis (Fig. 3A) and gene expression heatmaps (Fig. 3B) confirmed high reproducibility among parallel samples while revealing significant variations in cellular responses across stages. Differentially expressed genes (DEGs) were analyzed separately for each glutamate concentration, as shown in Fig. 3C. With 1 g/L glutamate, 645 DEGs (430 upregulated, 215 downregulated) were identified in GC1_4d vs. 2d, whereas only 104 DEGs (38 upregulated, 66 downregulated) were detected in GC1_6d vs. 4d. At 2 g/L glutamate, DEGs numbers increased substantially between fermentation stages: 939 DEGs (481 upregulated, 458 downregulated) in GC2_4d vs. 2d, and 1375 DEGs (751 upregulated, 624 downregulated) in GC2_6d vs. 4d.

**Fig. 4:**
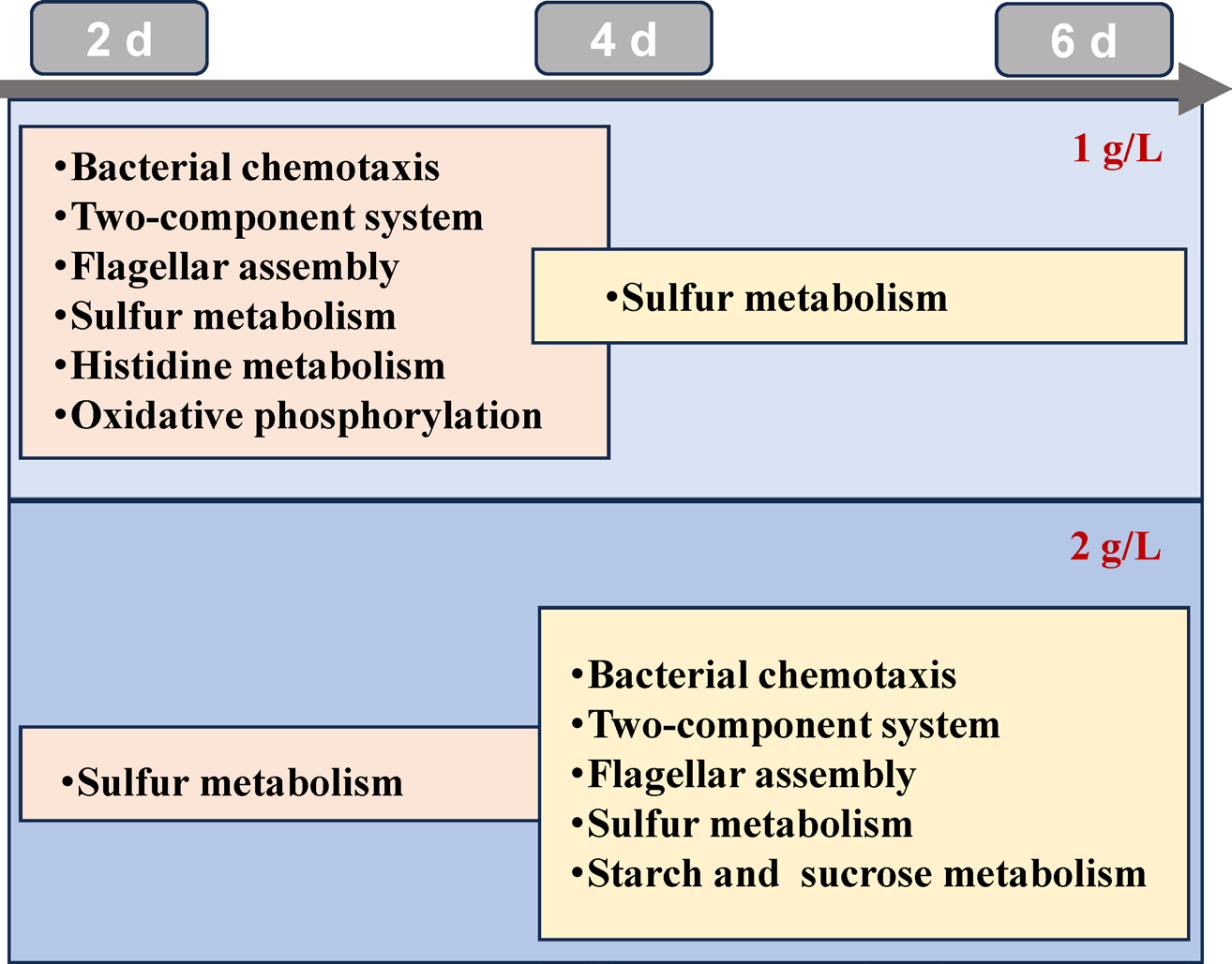
KEGG enrichment pathway of DEGs between different fermentation stages under 1 g/L and 2 g/L glutamate. Pathway framework adapted from KEGG database (www.kegg.jp/kegg/kegg1.html).

KEGG enrichment analysis (Fig. 4) revealed six significantly enriched pathways in GC1_4d vs. 2d, including bacterial chemotaxis, two-component system, flagellar assembly, sulfur metabolism, histidine metabolism, and oxidative phosphorylation. Notably, only sulfur metabolism remained differential expression in GC1_6d vs. 4d. Conversely, under 2 g/L glutamate conditions, sulfur metabolism was the exclusively enriched pathway in GC2_4d vs. 2d. In GC2_6d vs. 4d, starch and sucrose metabolism-related genes showed significantly upregulation, in addition to the first four pathways enriched in GC1_4d vs. 2d. This demonstrates that transcriptional responses were delayed in GC2 (day 6) relative to GC1 (day 4), correlating directly with initial glutamate concentration, which may act as the temporal regulator of metabolic remodeling.

In GC1_4d vs. 2d, genes involved in bacterial chemotaxis (40 genes) and flagellar assembly (35) were significantly upregulated (Table S1). These same genes exhibited conserved upregulation in GC2_6d vs. 4d (Table S4). Chemotaxis and motility play crucial roles in enhancing bacterial adaptability to nutrient fluctuations^25^, serving as conserved starvation response during nutrient depletion^26^. This process relies on the collaboration of chemotaxis proteins (MCP protein, sensing nutrient concentrations), signal transmission system (Che protein, connecting the flagella), and flagellum system (Fli, Flg, Mot, and Flh proteins, determining the direction of motion)^27^. Therefore, the time-shifted upregulation of these pathways (day 4 under 1 g/L and day 6 under 2 g/L) may demonstrate nitrogen limitation onset.

**Fig. 5:**
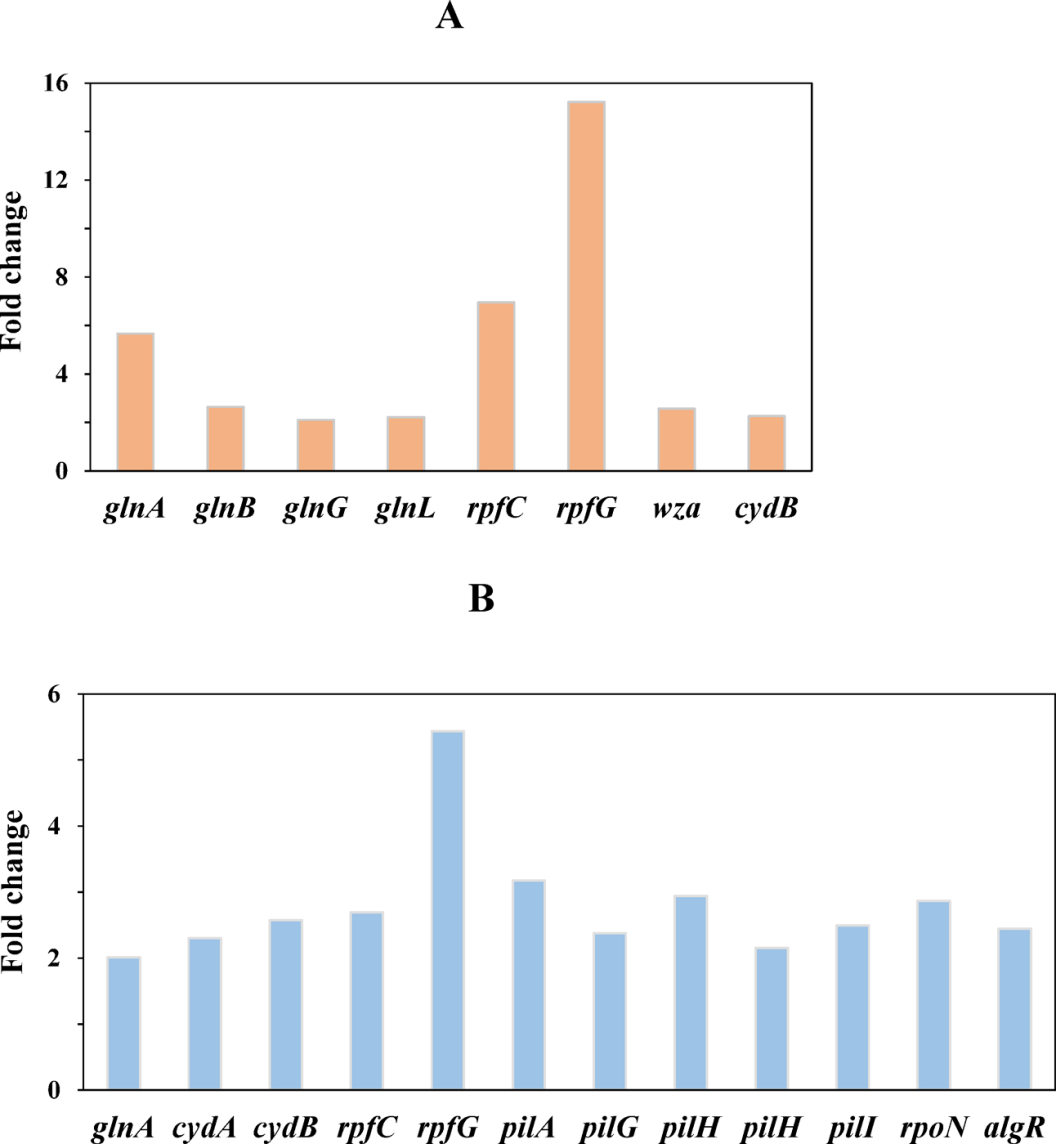
The fold change of DEGs in enriched pathway of two-component system. (**A**): GC1_4d vs. 2d; (**B**): GC2_6d vs. 4d; *glnA*: type I glutamate–ammonia ligase; *glnB*: nitrogen regulatory protein P-II 1; *glnG*: nitrogen regulation protein NR(I); *glnL*: signal transduction histidine kinase. *rpfC*: hybrid sensor histidine kinase/response regulator; *rpfG*: two-component system response regulator; *wza*: polysaccharide biosynthesis protein GumB; *cydA*: cytochrome d ubiquinol oxidase subunit I; *cydB*: cytochrome d ubiquinol oxidase subunit II; *pilA*: competence protein; *pilG*: pilus protein; *pilH*: two-component system response regulator protein; *pilH*: response regulator; *pilI*: pilus biogenesis protein; *rpoN*: RNA polymerase sigma-54 factor; *algR*: DNA-binding response regulator.

Among DGEs in the two-component system for GC1_4d vs. 2d (Fig. 5A and Table S1), *glnA*, *glnB*, *glnG* and *glnL* expression increased 5.67-, 2.65-, 2.11-, and 2.22-fold, respectively. The σ^54^-dependent *glnALG* operon plays a central role in nitrogen metabolism. GlnB (P_II_, a nitrogen regulatory protein) senses nitrogen limitation and subsequently stimulates the NtrB/NtrC system (encoded by *glnL*/*glnG*) and glutamine synthetase (*glnA*), facilitating conversion of glutamate to glutamine^28^. As primary nitrogen intermediates, glutamate and glutamine account for approximately 88% and 12% of cellular nitrogen, respectively^29,30^. As shown in Table S1, six *hut* operon gene (histidine metabolism) showed 2.18- to 4.64-fold upregulation, indicating enhanced glutamate production via histidine degradation^31^. Additionally, upregulated expression of oxidative phosphorylation genes (*cydB*, 2.28-fold) suggested elevated ATP demand under nitrogen limitation^28^. Collectively, these transcriptional changes demonstrated nitrogen assimilation reprogramming under N-limited conditions. Moreover, the two-component system encoded by *rpfC* (sensor kinase) and *rpfG* (response regulator), showed significant upregulation (*rpfC*, 6.95-fold; *rpfG*, 15.20-fold). In most *X. campestris* strains, the *rpf* cluster (*rpfC*, *rpfG*, *rpfB*, *rpfF*) is closely associated with diffusible signal factor (DSF)-mediated quorum sensing (QS), regulating population density, secondary metabolite biosynthesis, biofilm formation, and motility^32,33^. Critically, the RpfC-RpfG system governs DSF production through direct interaction with RpfF (DSF biosynthase), and positively regulates the xanthan gum biosynthesis in *X. campestris*^34,35^. Xanthan gum production in *Xanthomonas* is governed by the gum operon (12 genes: *gumB*-*gumM*). As shown in Table S2, only *gumB* (essential for xanthan polymerization and export) showed significant differential expression in GC1_4d vs. 2d, exhibiting a 2.58-fold increase. This limited transcriptional response suggested minimal glutamate concentration effects on the *gum* operon. Galván et al. demonstrated that GumB–GumC complexes modulate xanthan chain length, thereby affecting polymer viscosity^36^. Therefore, *gumB* overexpression likely contributed to the enhanced xanthan yield and viscosity observed on day 4 under 1 g/L glutamate. Within the DEGs of the two-component system in GC2_6d vs. 4d (Fig. 5B and Table S4), *gln* gene (*glnA*), respiratory chain genes (*cydA* and *cydB*), and *rpf* genes (*rpfC* and *rpfG*) were overexpressed. Furthermore, *pilAGHI* (type IV pilus, T4P), *algR* (transcriptional response regulator), and *rpoN* (Sigma factor 54, σ^54^) were up-regulated 2.16- to 3.17-, 2.45-, and 2.87-fold, respectively. Type IV pilus in *Xanthomonas* spp. play important roles in motility, biofilm formation, and plant pathogenesis^37^. It has been reported that AlgR involved in regulating the motility and EPS (rhamnolipid) production in *Pseudomonas aeruginosa*^38^. The transcriptional regulator rpoN is well-known for its role in mediating bacterial adaptation to environmental stress, influencing nitrogen assimilation, growth, flagella assembly, EPS formation, biofilm formation, QS, amino acid utilization, and carbohydrate metabolism^39,40^. Collectively, this expression profile suggested glutamate limitation under 2 g/L concentration on day 6.

Regarding sulfur metabolism, significant upregulation occurred in genes for extracellular sulfur uptake (*tau* and *ssu* system), cysteine biosynthesis (*cys* operon), and methionine biosynthesis (*metX*/*metB*) both in GC1_4d vs. 2d (Table S1) and GC2_4d vs. 2d (Table S3)^41^. Notably, the expression of *tauD* gene (encoding taurine dioxygenase for converting taurine to sulfite and aminoacetaldehyde) increased 16.39- and 7.24-fold in these comparisons, respectively. Sulfur-containing amino acids are essential components of proteins across organisms. Subsequently, sulfur uptake gene expression decreased in GC1_6d vs. 4d (Table S3) and GC2_6d vs. 4d (Table S4), with all sulfur metabolism DEGs (including *cys*, *ssu*, and *tau* genes) downregulated 2.18- to 25-fold in the latter comparison^41^. This suppression likely reflected carbon flux toward xanthan gum biosynthesis and secretion during stationary phases. In summary, glutamate concentration governed nitrogen limitation timing during xanthan gum production in *X. campestris*. With 1 g/L and 2 g/L nitrogen sources, nitrogen limitation occurred on day 4 and day 6, respectively. Under N-limited conditions, strain E01 activated chemotaxis, flagellar assembly, glutamine synthesis, and respiratory chain pathways to enhance nitrogen assimilation, while simultaneously regulating DSF synthesis to promote higher yields and viscosity of xanthan gum. These adaptive responses highlight the advantageous role of N limitation in optimizing xanthan gum production.

### Transcriptomic analysis under different glutamate concentrations

**Fig. 6:**
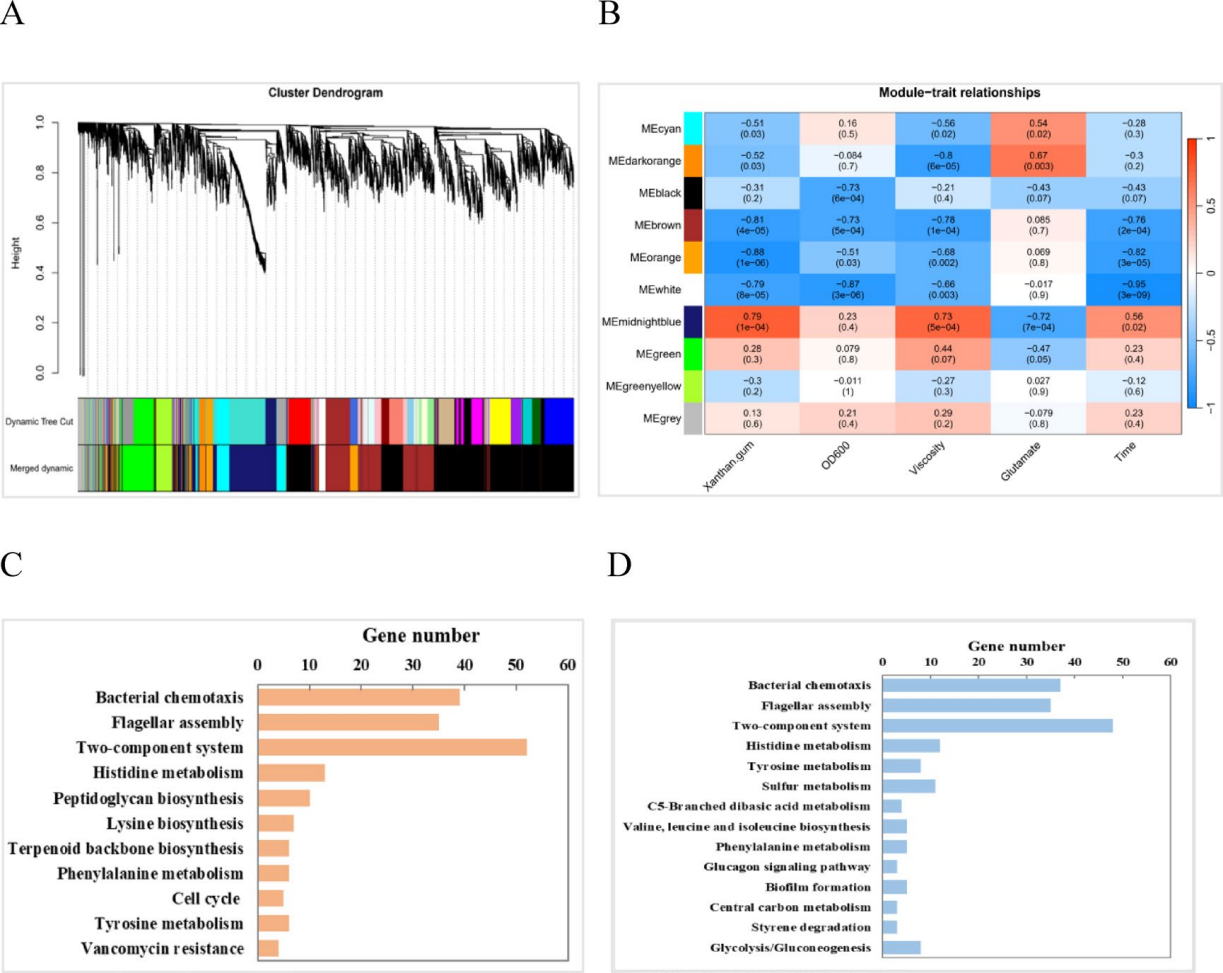
Weighted gene co-expression network analysis (WGCNA) of all DEGs. (**A**): Hierarchical clustering trees and modules. Different colors represent different modules. (**B**): Module-trait relationships. Red and blue represent positive and negative correlations, respectively. (**C**): Number of genes in KEGG enrichment pathways for  4d_GC2 vs. GC1. (**D**): Number of genes in KEGG enrichment pathways for 6d_GC2 vs. GC1.

To identify the key genes influencing yield and viscosity of xanthan gum, WGCNA was used to access the relationships between DEGs and the corresponding phenotypes. Highly correlated gene clusters were grouped into modules, resulting in nine different modules following network construction (Fig. 6A). Among them, five modules (namely brown, midnightblue, orange, darkorange, and white) exhibited significant positive correlations (correlation coefficients > 0.7) with either yield or viscosity of xanthan gum (Fig. 6B). Consequently, all 318 genes from these five modules underwent further analysis using KEGG pathway enrichment.

Expression profiles of DEGs in enriched KEGG pathways for the 4d_GC2 vs. GC1 and 6d_GC2 vs. GC1 comparisons are presented in Table S5 and Table S6, respectively. Among the 11 pathways significantly enriched in 4d_GC2 vs. GC1 (Fig. 6C), 39 genes in bacterial chemotaxis and 35 genes in flagellar assembly were significantly down-regulated. In the two-component system pathway, expression of *cydA*, *cydB*, *rpfC*,* rpfG*, and *rpoN* was depressed by 2.17-fold, 2.32-fold, 25-fold, 9.09-fold, and 4.76-fold, respectively, while *glnD* exhibited a 3.26-fold upregulation. Furthermore, five cell cycle-related genes (*ftsA*, *ftsQ*, *ftsZ*, *ftsW*, and *murG*) displayed significantly upregulation. Concurrently, ten genes associated with peptidoglycan biosynthesis (*ddl*, *ftsI*, *mraY*, *mrcA*, *dacC*, *bcrC*, *murC*, *murG*, *murE*, and *murF*) exhibited increased expression, consistent with peptidoglycan’s role as a critical bacterial cell wall component^42^. Additionally, three genes (*dapD*, *dapE*,* dapF*) involved in lysine biosynthesis from aspartate were upregulated (2.22 to 2.88- fold changes). This lysine upregulation is functionally significant, as lysine cross-links glycan chains via the MurE-MurF complex, thereby enhancing peptidoglycan structural rigidity^43^. This molecular profile aligned with the enhanced cell growth observed on day 4 under 2 g/L glutamate, as shown in the Fig. 1. Li et al. reported that inhibiting peptidoglycan biosynthesis with ampicillin stress increased productivity and viscosity of xanthan gum in *X. campestris*^44^. Given that peptidoglycan biosynthesis shares initial metabolic precursors with xanthan gum production, we hypothesize that enhanced cell growth may redirect carbon flux away from xanthan gum synthesis, potentially reducing both yield and viscosity.

In 6d_GC2 vs. GC1 (Fig. 6D), 16 pathways were significantly enriched by KEGG analysis. Similarly, all DEGs associated with bacterial chemotaxis and flagellar assembly were downregulated (Table S6). Within the two-component system, *rpoN*, *rpfC* and *rpfG* exhibited reduced expression (2.13-, 2.63-, and 7.14-fold, respectively), while *cydB* was upregulated 7.78-fold. Furthermore, genes involved in histidine metabolism (biosynthesis and degradation), sulfur metabolism, and biofilm formation also showed downregulation. Specifically, four biofilm-related genes (*oxyR*, *flgM*, *fliA*, *bcsA*) displayed 6.67-, 3.33-, 5.26-, and 3.45-fold depressed expression. In *X. campestris*, xanthan gum constitutes a critical component of the biofilm extracellular matrix^45^. The suppression of these genes likely contributed to the yield and viscosity decline on day 6 under 2 g/L glutamate. Conversely, seven genes (*gpmA*, *tpiA*, *pdhA*, *pdhB*, *aceF*, *adhP* and *aldB*) involved in Glycolysis, Central carbon metabolism, and Glucagon signaling were upregulated (2.04- to 10.41-fold). Among them, GpmA (phosphoglycerate mutase) and TpiA (triosephosphate isomerase), three pyruvate dehydrogenases (PdhA, PdhB, and AceF), AdhP (alcohol dehydrogenase) and AldB (acetaldehyde dehydrogenase), are central to the conversion of glucose to ethanol. Additionally, pathway enrichment also included C5-Branched dibasic acid metabolism and branched chain-amino acid (Valine, leucine, and isoleucine) biosynthesis. It has been reported that  *X*. *campestris* could produce branched chain fatty acids (BCFAs) accounting for approximately 50% of the total cellular fatty acids^46^. BCFAs have been well-known as the important component of DSF-family signals, which govern pathogenesis, biofilm formation, EPS production, and motility^46,47^. As precursors of BCFA, three branched-chain 2-keto acids are derived from carbohydrate metabolism, including 2-keto-methylvalerate (KMV), 2-ketoisovalerate (KIV) and 2-ketoisocaproate (KIC). Among them, *ilvGMCD* are responsible for KMV and KIV production from pyruvate, while *leuABCD* convert KIV into KIC^48^. In the 6d_GC2 vs. GC1, *ilvB* and *ilvC* expression decreased 4.00-fold and 5.56-fold, respectively, while *leuB*, *leuC* and *leuD* increased 2.38-, 3.79-, and 3.17-fold, this opposing regulation suggests compromised BCFA biosynthesis. According to the reports from Li et al.^48^, an *ilvC* mutant disrupted DSF-family signal production (particularly branched-chain type), attenuating pathogenesis in *Xcc* strain. Moreover, Δ*rpoN* mutant studies confirmed RpoN’s regulatory role in BCFA and DSF-family signals biosynthesis^39^.

**Fig. 7:**
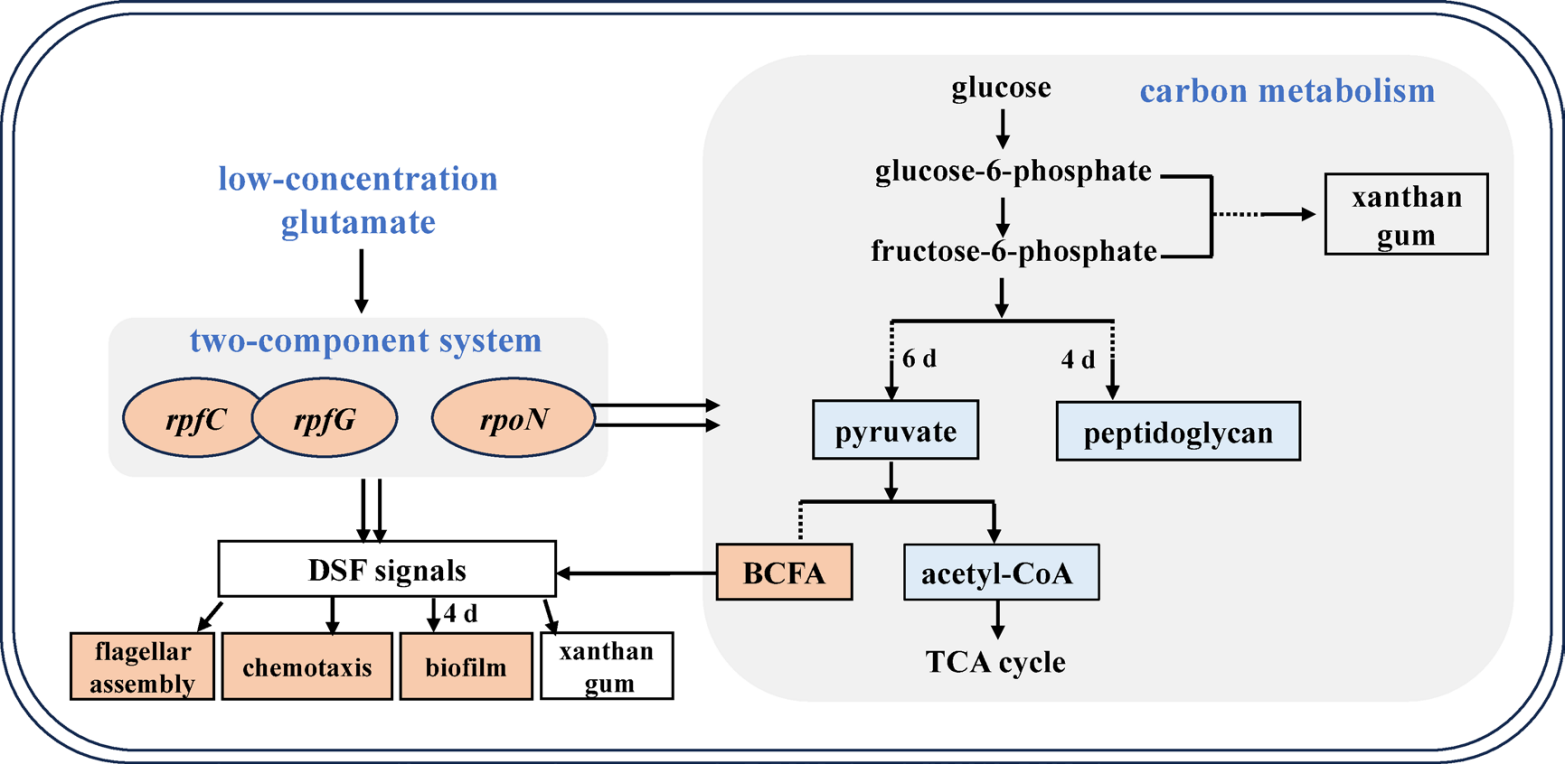
Transcriptional mechanism of glutamate concentration on xanthan gum. Orange: up-regulated; blue: down-regulated.

Based on these findings, we propose a transcriptional mechanism for glutamate concentration-dependent regulation of xanthan gum production (Fig. 7). At low glutamate concentration (1 g/L), upregulation of *rpoN* enhances nitrogen assimilation through increased bacterial chemotaxis and flagellar assembly, facilitating nutrient uptake. Concurrently, carbon flux was redirected away from peptidoglycan biosynthesis on day 4 and central carbon metabolism (pyruvate/acetyl-CoA biosynthesis) on day 6, thereby enhancing xanthan gum synthesis. Additionally, elevated BCFA biosynthesis from pyruvate may stimulate DSF-family signal production, which regulates multiple cellular processes including motility and EPS biosynthesis. The upregulated *rpfC-rpfG* regulatory system under 1 g/L glutamate further promotes xanthan gum production through DSF signal modulation. Collectively, low glutamate concentration promotes xanthan gum biosynthesis primarily by coordinating two-component systems: the *rpoN*-mediated metabolic pathway and the *rpfC-rpfG* regulatory cascade.

Taking together, glutamate demonstrates significant potential as an effective nitrogen source for xanthan gum biosynthesis by *X. campestris*. This study revealed that low nitrogen concentration enhanced both the yield and viscosity of xanthan gum. These improvements are attributed to upregulated expression of two-component regulatory system genes, which optimize nitrogen assimilation and redirect carbon fluxes toward xanthan gum production. Further functional validation of these key genes is warranted to elucidate their precise roles in xanthan gum biosynthesis. Such efforts would accelerate the development of engineered strains for xanthan gum production, thereby advancing industry applications.

## Methods

### Strain and media

The bacterial strain *Xanthomonas campestris* E01 (GIM1.857) was purchased from Guangdong Microbial Culture Collection Center in China. An LB plate (10 g/L peptone, 5 g/L yeast extract, 10 g/L NaCl, 20 g/L agar, pH 7.0) was used for strain activation. For pre-cultivation, LB medium was employed. Batch fermentation was carried out using a medium consisting of 40 g/L glucose, 1 g/L or 2 g/L of a nitrogen source (NH_4_Cl or glutamate), 3 g/L CaCO_3_, 5 g/L KH_2_PO_4_, 0.5 g/L MgSO_4_·7H_2_O, 0.25 g/L FeSO_4_, and 1 g/L citric acid (pH 7.0).

### Batch fermentation and analytical methods

After activation, cells were inoculated into a 500-mL Erlenmeyer flask containing 100 mL of LB medium and cultivated at 30 °C with shaking at 120 rpm for 24 h. A 40 mL aliquot of this culture was then transferred to a 1 L flask containing 400 mL of the fermentation medium. Fermentation was conducted at 30 °C with orbital shaking at 180 rpm for 6 days. All fermentation experiments were performed independently in triplicate.

Samples were collected daily to analyze cell growth, broth viscosity, residual glucose concentration, and xanthan gum yield. Cell biomass was determined by measuring the optical density at 660 nm (OD₆₆₀) using a spectrophotometer (V-530, JASCO, Japan). Broth viscosity was measured at 25 °C with a rotational viscometer (TVB-10, TOKISANGYO, Japan) equipped with rotor No. M2 at 60 rpm. For glucose and xanthan gum analysis, samples were first centrifuged at 10,000 × g for 3 min at 25 °C, and the supernatant was filtered through a 0.45-µm membrane filter. Residual glucose concentration was quantified by high-performance liquid chromatography (HPLC; Aminex HPX-87 H column, Bio-Rad, USA) using 500 mM H₂SO₄ as the mobile phase at a flow rate of 0.8 mL/min. Xanthan gum yield was determined by mixing 10 mL of the supernatant with 30 mL of absolute ethanol. The resulting precipitate was collected by filtration, dried at 105 °C overnight, and weighed to calculate the xanthan gum yield. Data are presented as the mean ± standard deviation from three independent experiments. Statistical significance was assessed by an independent samples *t*-test.

### RNA extraction and sequencing

Cells were collected at multiple time points (days 2, 4, and 6) during fermentation under two nitrogen conditions: with 1 g/L glutamate (designated GC1) and 2 g/L glutamate (GC2). Total RNA was extracted using TRIzol Reagent (Invitrogen, Thermo Fisher Scientific, USA) according to the manufacturer’s instructions. RNA quality and concentration were assessed using an Agilent 2100 Bioanalyzer (Agilent Technologies, USA). Ribosomal RNA was depleted using the Ribo-Zero Magnetic Kit (Epicentre, USA) to enrich messenger RNA (mRNA). The purified mRNA was randomly fragmented and then reverse-transcribed into double-stranded cDNA using random primers and the SuperScript Double-Stranded cDNA Synthesis Kit (Invitrogen, USA). The resulting cDNA libraries were constructed through end repair, poly(A) tailing, and adapter ligation, and subsequently sequenced on an Illumina HiSeq platform (Illumina, USA) by Majorbio Bio-Pharm Technology Co., Ltd. (Shanghai, China).

### Transcriptomic analysis

After filtering low-quality raw reads, the remaining reads were aligned to the *X. campestris* reference genome using Bowtie2 (v2.x) software^49^. Gene expression levels were quantified in Fragments Per Kilobase of transcript per Million mapped reads (FPKM) through the RSEM software. Differentially expressed genes (DEGs) were identified with DESeq2 (http://bioconductor.org/packages/release/bioc/html/DESeq2.html) using thresholds of |log_2_(fold change) |≥1 and *P* < 0.05. Kyoto Encyclopedia of Genes and Genomes (KEGG) enrichment analysis^50–52^ of the DEGs was performed using KEGG Orthology Based Annotation System (KOBAS) (http://kobas.cbi.pku.edu.cn). KEGG pathways with a *p*_*adj*_ < 0.05 were considered to be significantly enriched.

Weighted Gene Co-Expression Network Analysis (WGCNA) was conducted on 18 RNA samples using the WGCNA package in R^53^. The procedure comprised three main stages. First, gene expression data were normalized using log_2_(FPKM + 1), and sample clustering was performed to detect and remove outliers. Second, a co-expression network was constructed. A suitable soft-thresholding (13) was selected to generate a topological overlap matrix (TOM). Based on pairwise Pearson correlations among all genes, a correlation matrix was established. Hierarchical clustering was applied to the TOM matrix to identify gene modules with similar expression profiles, and the DynamicTreeCut algorithm was employed to merge modules exhibiting comparable expression patterns. Third, module-trait associations were analyzed. Principal component analysis (PCA) was performed on the expression matrix of each module, and the first principal component (module eigengene, ME) was extracted to represent its overall expression pattern. Correlation between the MEs and the sample traits (or conditions) was then calculated to identify which gene modules were most strongly associated with the specific treatments of interest, thereby facilitating the selection of key modules for further investigation.

## Supplementary Information

Below is the link to the electronic supplementary material.

Supplementary Material 1

## Data Availability

The transcriptome datasets analyzed in this study can be accessed through the SRA accession PRJNA1121488.

## Abbreviations

EPS: Exopolysaccharides
DEGs: Differentially expressed genes
WGCNA: Weighted gene co-expression network analysis
DSF: Diffusible signal factor
QS: Quorum sensing
BCFAs: Branched chain fatty acids
KMV: 2-keto-methylvalerate
KIV: 2-ketoisovalerate
KIC: 2-ketoisocaproate
