# Supplementary material for: Transcriptomic response of Xanthomonas campestris during xanthan gum production to glutamate concentration
Source: Sci Rep. 2026 Mar 13;16:13377. doi: 10.1038/s41598-026-43665-8 (PMC13106696; doi:10.1038/s41598-026-43665-8)
Supplement: Supplementary file 1 — Supplementary Material 1 [file 41598_2026_43665_MOESM1_ESM.docx]

**Transcriptomic response of *Xanthomonas* *campestris* during xanthan gum production to glutamate concentration**

Lu Wang^a,b^, Xinmin Song^a,b^, Chuanfu Ji^c^, Maozhang Tian^a,b^, Caiyun Xie^c,*^, Min Gou^c,*^, Wenfeng Song^b^, Yueqin Tang^c^

*^a^ State Key Laboratory of Enhanced Oil & Gas Recovery, Beijing 100083, China*

*^b^ Research Institute of Petroleum Exploration & Development, CNPC, Beijing 100083, China*

*^c^ College of Architecture and Environment, Sichuan University, Chengdu, 610065, China*

*Corresponding author:

Caiyun Xie, Tel. +86(28)85990936, E-mail. [xiecy@scu.edu.cn](mailto:xiecy@scu.edu.cn)

Min Gou, Tel. +86(28)85990936, E-mail. [goumin@scu.edu.cn](mailto:goumin@scu.edu.cn)

*College of Architecture and Environment, Sichuan University, No. 24 South Section 1 First Ring Road, Chengdu, 610065, China*

**Table S1** **The expression of DEGs in KEGG enrichment pathways of GC1_4d vs 2d**

| **Pathway** | **Gene_id** | **Gene name** | **Gene description** | **FC(G1_4/G1_2)** | **Log2FC(G1_4/G1_2)** | **Regulate** |
| --- | --- | --- | --- | --- | --- | --- |
| Bacterial chemotaxis | GeFixPreAbcdefg2157 | cheW | chemotaxis protein | 23.39 | 4.547839851 | up |
|  | GeFixPreAbcdefg2190 | cheZ | chemotaxis protein | 29.857 | 4.899997692 | up |
|  | GeFixPreAbcdefg2191 | cheY | response regulator | 29.728 | 4.893765617 | up |
|  | GeFixPreAbcdefg2189 | cheA | chemotaxis protein CheA | 25.576 | 4.67673795 | up |
|  | GeFixPreAbcdefg2572 | cheW | hypothetical protein | 55.471 | 5.793672331 | up |
|  | GeFixPreAbcdefg2154 | cheA | chemotaxis protein CheA | 21.441 | 4.4222727 | up |
|  | GeFixPreAbcdefg2979 | cheW | hypothetical protein | 13.898 | 3.796761769 | up |
|  | GeFixPreAbcdefg2135 | cheW | chemotaxis protein CheW | 32.135 | 5.006083533 | up |
|  | GeFixPreAbcdefg2254 | cheV | conserved hypothetical protein | 30.337 | 4.922994915 | up |
|  | GeFixPreAbcdefg2155 | cheY | Fis family transcriptional regulator | 20.865 | 4.383043026 | up |
|  | GeFixPreAbcdefg2977 | cheA | chemotaxis protein CheA | 29.781 | 4.896338466 | up |
|  | GeFixPreAbcdefg2132 | cheR | chemotaxis protein CheR | 23.909 | 4.579466626 | up |
|  | GeFixPreAbcdefg2981 | cheR | SAM-dependent methyltransferase | 9.703 | 3.278459736 | up |
|  | GeFixPreAbcdefg2982 | cheB | chemotaxis response regulator protein-glutamate methylesterase | 3.463 | 1.791814119 | up |
|  | GeFixPreAbcdefg2130 | cheB | chemotaxis response regulator protein-glutamate methylesterase | 2.48 | 1.310085864 | up |
|  | GeFixPreAbcdefg2573 | mcp | chemotaxis protein | 64.944 | 6.021114892 | up |
|  | GeFixPreAbcdefg2978 | mcp | chemotaxis protein | 20.986 | 4.391335881 | up |
|  | GeFixPreAbcdefg2148 | mcp | chemotaxis protein | 32.659 | 5.029430768 | up |
|  | GeFixPreAbcdefg2143 | mcp | chemotaxis protein | 21.837 | 4.448711961 | up |
|  | GeFixPreAbcdefg3354 | mcp | chemotaxis protein | 15.685 | 3.971299628 | up |
|  | GeFixPreAbcdefg2137 | mcp | chemotaxis protein | 28.874 | 4.851690646 | up |
|  | GeFixPreAbcdefg2263 | mcp | methyl-accepting chemotaxis (MCP) signaling domain protein | 28.452 | 4.830451055 | up |
|  | GeFixPreAbcdefg2147 | mcp | chemotaxis protein | 15.385 | 3.943469661 | up |
|  | GeFixPreAbcdefg1981 | mcp | chemotaxis protein | 9.483 | 3.245368554 | up |
|  | GeFixPreAbcdefg2133 | mcp | chemotaxis protein | 10.488 | 3.390657531 | up |
|  | GeFixPreAbcdefg2138 | mcp | chemotaxis protein | 2.28 | 1.188938581 | up |
|  | GeFixPreAbcdefg3565 | tar | chemotaxis protein | 3.159 | 1.659366565 | up |
|  | GeFixPreAbcdefg2219 | fliG | flagellar motor switch protein FliG | 17.577 | 4.13560497 | up |
|  | GeFixPreAbcdefg2211 | fliNY | flagellar motor switch protein FliN | 13.068 | 3.707970138 | up |
|  | GeFixPreAbcdefg2212 | fliM | flagellar motor switch protein FliM | 16.554 | 4.049134995 | up |
|  | GeFixPreAbcdefg2159 | motB | flagellar motor protein MotD | 32.486 | 5.021755035 | up |
|  | GeFixPreAbcdefg2160 | motA | flagellar motor protein | 24.21 | 4.597502285 | up |
|  | GeFixPreAbcdefg3799 | motB | flagellar motor protein MotB | 16.084 | 4.007516698 | up |
|  | GeFixPreAbcdefg3798 | motA | flagellar motor protein MotA | 8.477 | 3.083508584 | up |
|  | GeFixPreAbcdefg2144 | - | chemotaxis protein | 27.225 | 4.766841656 | up |
|  | GeFixPreAbcdefg2145 | - | chemotaxis protein | 26.079 | 4.704826739 | up |
|  | GeFixPreAbcdefg1527 | - | chemotaxis protein | 33.647 | 5.072393259 | up |
|  | GeFixPreAbcdefg2153 | - | chemotaxis protein | 25.441 | 4.669064929 | up |
|  | GeFixPreAbcdefg2151 | - | chemotaxis protein | 7.188 | 2.845518186 | up |
|  | GeFixPreAbcdefg2140 | - | chemotaxis protein | 10.076 | 3.332812535 | up |
| Flagellar assembly | GeFixPreAbcdefg2246 | flgH | flagellar basal body L-ring protein | 26.732 | 4.740499793 | up |
|  | GeFixPreAbcdefg2201 | flhA | flagellar biosynthesis protein FlhA | 21.11 | 4.399825983 | up |
|  | GeFixPreAbcdefg2220 | fliF | flagellar M-ring protein FliF | 22.557 | 4.495486113 | up |
|  | GeFixPreAbcdefg2243 | flgK | flagellar hook protein FlgK | 25.992 | 4.700007559 | up |
|  | GeFixPreAbcdefg2253 | flgB | flagellar biosynthesis protein FlgB | 30.738 | 4.941958415 | up |
|  | GeFixPreAbcdefg2159 | motB | flagellar motor protein MotD | 32.486 | 5.021755035 | up |
|  | GeFixPreAbcdefg2247 | flgG | flagellar basal body rod protein FlgG | 29.56 | 4.885553526 | up |
|  | GeFixPreAbcdefg2245 | flgI | flagellar P-ring protein | 22.223 | 4.473999098 | up |
|  | GeFixPreAbcdefg2210 | fliOZ | flagellar biosynthetic protein FliO | 17.061 | 4.092592498 | up |
|  | GeFixPreAbcdefg2242 | flgL | flagellar hook protein FlgL | 27.217 | 4.76643752 | up |
|  | GeFixPreAbcdefg2249 | flgF | flagellar basal body rod protein FlgF | 30.311 | 4.921763561 | up |
|  | GeFixPreAbcdefg2202 | flhB | flagellar biosynthesis protein FlhB | 24.13 | 4.592782322 | up |
|  | GeFixPreAbcdefg2219 | fliG | flagellar motor switch protein FliG | 17.577 | 4.13560497 | up |
|  | GeFixPreAbcdefg2207 | fliR | flagellar biosynthetic protein FliR | 19.614 | 4.29379903 | up |
|  | GeFixPreAbcdefg2256 | flgM | flagellar biosynthesis anti-sigma factor FlgM | 24.332 | 4.604801273 | up |
|  | GeFixPreAbcdefg2255 | flgA | flagellar basal body P-ring biosynthesis protein FlgA | 31.579 | 4.980897208 | up |
|  | GeFixPreAbcdefg2221 | fliE | flagellar hook-basal body protein | 25.065 | 4.64759839 | up |
|  | GeFixPreAbcdefg2160 | motA | flagellar motor protein | 24.21 | 4.597502285 | up |
|  | GeFixPreAbcdefg2216 | fliJ | flagellar export protein FliJ | 15.185 | 3.924560117 | up |
|  | GeFixPreAbcdefg2251 | flgD | flagellar basal body rod modification protein | 25.926 | 4.696318429 | up |
|  | GeFixPreAbcdefg2252 | flgC | flagellar basal body rod protein FlgC | 27.836 | 4.798880624 | up |
|  | GeFixPreAbcdefg2240 | fliD | flagellar protein | 20.687 | 4.370658042 | up |
|  | GeFixPreAbcdefg2192 | fliA | RNA polymerase sigma factor FliA | 30.059 | 4.90973804 | up |
|  | GeFixPreAbcdefg2215 | fliK | flagellar protein | 15.392 | 3.94414569 | up |
|  | GeFixPreAbcdefg3799 | motB | flagellar motor protein MotB | 16.084 | 4.007516698 | up |
|  | GeFixPreAbcdefg2209 | fliP | flagellar biosynthetic protein FliP | 14.137 | 3.821366606 | up |
|  | GeFixPreAbcdefg2250 | flgE | flagellar hook protein FlgE | 28.107 | 4.812842111 | up |
|  | GeFixPreAbcdefg2208 | fliQ | flagellar biosynthesis | 21.204 | 4.406231082 | up |
|  | GeFixPreAbcdefg2241 | fliC | flagellin | 31.15 | 4.961166127 | up |
|  | GeFixPreAbcdefg2211 | fliNY | flagellar motor switch protein FliN | 13.068 | 3.707970138 | up |
|  | GeFixPreAbcdefg2239 | fliS | flagellar protein FliS | 24.049 | 4.587914163 | up |
|  | GeFixPreAbcdefg2217 | fliI | flagellar protein export ATPase FliI | 15.324 | 3.937683195 | up |
|  | GeFixPreAbcdefg2212 | fliM | flagellar motor switch protein FliM | 16.554 | 4.049134995 | up |
|  | GeFixPreAbcdefg3798 | motA | flagellar motor protein MotA | 8.477 | 3.083508584 | up |
|  | GeFixPreAbcdefg2218 | fliH | flagellar assembly protein FliH | 14.661 | 3.873900612 | up |
| Two-component system | GeFixPreAbcdefg0030 | - | cellulase | 201.876 | 7.657329036 | up |
|  | GeFixPreAbcdefg0031 | - | cellulase | 3.868 | 1.95174653 | up |
|  | GeFixPreAbcdefg2144 | - | chemotaxis protein | 27.225 | 4.766841656 | up |
|  | GeFixPreAbcdefg2145 | - | chemotaxis protein | 26.079 | 4.704826739 | up |
|  | GeFixPreAbcdefg1527 | - | chemotaxis protein | 33.647 | 5.072393259 | up |
|  | GeFixPreAbcdefg2153 | - | chemotaxis protein | 25.441 | 4.669064929 | up |
|  | GeFixPreAbcdefg2151 | - | chemotaxis protein | 7.188 | 2.845518186 | up |
|  | GeFixPreAbcdefg2140 | - | chemotaxis protein | 10.076 | 3.332812535 | up |
|  | GeFixPreAbcdefg2489 | - | putative membrane protein | 0.093 | -3.425098866 | down |
|  | GeFixPreAbcdefg0778 | - | endoglucanase | 3.959 | 1.985139397 | up |
|  | GeFixPreAbcdefg0132 | - | pectin esterase | 2.142 | 1.098893567 | up |
|  | GeFixPreAbcdefg3193 | ampC | serine hydrolase | 3.249 | 1.700050759 | up |
|  | GeFixPreAbcdefg3403 | cbhA | 1,4-beta-cellobiosidase | 4.228 | 2.080095488 | up |
|  | GeFixPreAbcdefg2189 | cheA | chemotaxis protein CheA | 25.576 | 4.67673795 | up |
|  | GeFixPreAbcdefg2154 | cheA | chemotaxis protein CheA | 21.441 | 4.4222727 | up |
|  | GeFixPreAbcdefg2977 | cheA | chemotaxis protein CheA | 29.781 | 4.896338466 | up |
|  | GeFixPreAbcdefg2982 | cheB | chemotaxis response regulator protein-glutamate methylesterase | 3.463 | 1.791814119 | up |
|  | GeFixPreAbcdefg2130 | cheB | chemotaxis response regulator protein-glutamate methylesterase | 2.48 | 1.310085864 | up |
|  | GeFixPreAbcdefg2132 | cheR | chemotaxis protein CheR | 23.909 | 4.579466626 | up |
|  | GeFixPreAbcdefg2981 | cheR | SAM-dependent methyltransferase | 9.703 | 3.278459736 | up |
|  | GeFixPreAbcdefg2254 | cheV | conserved hypothetical protein | 30.337 | 4.922994915 | up |
|  | GeFixPreAbcdefg2157 | cheW | chemotaxis protein | 23.39 | 4.547839851 | up |
|  | GeFixPreAbcdefg2572 | cheW | hypothetical protein | 55.471 | 5.793672331 | up |
|  | GeFixPreAbcdefg2979 | cheW | hypothetical protein | 13.898 | 3.796761769 | up |
|  | GeFixPreAbcdefg2135 | cheW | chemotaxis protein CheW | 32.135 | 5.006083533 | up |
|  | GeFixPreAbcdefg2191 | cheY | response regulator | 29.728 | 4.893765617 | up |
|  | GeFixPreAbcdefg2155 | cheY | Fis family transcriptional regulator | 20.865 | 4.383043026 | up |
|  | GeFixPreAbcdefg2487 | cydA | cytochrome d ubiquinol oxidase, subunit II | 0.056 | -4.146212098 | down |
|  | GeFixPreAbcdefg2488 | cydB | cytochrome d ubiquinol oxidase subunit II | 0.094 | -3.40692718 | down |
|  | GeFixPreAbcdefg3110 | cydB | cytochrome d ubiquinol oxidase subunit II | 0.415 | -1.26931684 | down |
|  | GeFixPreAbcdefg3844 | cydB | cytochrome d ubiquinol oxidase subunit II | 2.268 | 1.181701735 | up |
|  | GeFixPreAbcdefg4101 | desR | DNA-binding response regulator | 2.322 | 1.215354689 | up |
|  | GeFixPreAbcdefg2256 | flgM | flagellar biosynthesis anti-sigma factor FlgM | 24.332 | 4.604801273 | up |
|  | GeFixPreAbcdefg2192 | fliA | RNA polymerase sigma factor FliA | 30.059 | 4.90973804 | up |
|  | GeFixPreAbcdefg2241 | fliC | flagellin | 31.15 | 4.961166127 | up |
|  | GeFixPreAbcdefg0209 | glnA | type I glutamate--ammonia ligase | 5.667 | 2.50265012 | up |
|  | GeFixPreAbcdefg0210 | glnB | nitrogen regulatory protein P-II 1 | 2.654 | 1.407981043 | up |
|  | GeFixPreAbcdefg0214 | glnG | nitrogen regulation protein NR(I) | 2.111 | 1.078174856 | up |
|  | GeFixPreAbcdefg0213 | glnL | signal transduction histidine kinase | 2.224 | 1.15301445 | up |
|  | GeFixPreAbcdefg0966 | kdpA | potassium-transporting ATPase subunit KdpA | 3.84 | 1.94100258 | up |
|  | GeFixPreAbcdefg0967 | kdpB | K+-transporting ATPase subunit B | 3.151 | 1.655940729 | up |
|  | GeFixPreAbcdefg2573 | mcp | chemotaxis protein | 64.944 | 6.021114892 | up |
|  | GeFixPreAbcdefg2978 | mcp | chemotaxis protein | 20.986 | 4.391335881 | up |
|  | GeFixPreAbcdefg2148 | mcp | chemotaxis protein | 32.659 | 5.029430768 | up |
|  | GeFixPreAbcdefg2143 | mcp | chemotaxis protein | 21.837 | 4.448711961 | up |
|  | GeFixPreAbcdefg3354 | mcp | chemotaxis protein | 15.685 | 3.971299628 | up |
|  | GeFixPreAbcdefg2137 | mcp | chemotaxis protein | 28.874 | 4.851690646 | up |
|  | GeFixPreAbcdefg2263 | mcp | methyl-accepting chemotaxis (MCP) signaling domain protein | 28.452 | 4.830451055 | up |
|  | GeFixPreAbcdefg2147 | mcp | chemotaxis protein | 15.385 | 3.943469661 | up |
|  | GeFixPreAbcdefg1981 | mcp | chemotaxis protein | 9.483 | 3.245368554 | up |
|  | GeFixPreAbcdefg2133 | mcp | chemotaxis protein | 10.488 | 3.390657531 | up |
|  | GeFixPreAbcdefg2138 | mcp | chemotaxis protein | 2.28 | 1.188938581 | up |
|  | GeFixPreAbcdefg3974 | mdtA | efflux transporter periplasmic adaptor subunit | 2.006 | 1.004377427 | up |
|  | GeFixPreAbcdefg2160 | motA | flagellar motor protein | 24.21 | 4.597502285 | up |
|  | GeFixPreAbcdefg3798 | motA | flagellar motor protein MotA | 8.477 | 3.083508584 | up |
|  | GeFixPreAbcdefg2118 | rpfC | hybrid sensor histidine kinase/response regulator | 6.953 | 2.797718815 | up |
|  | GeFixPreAbcdefg2116 | rpfG | two-component system response regulator | 15.209 | 3.926855336 | up |
|  | GeFixPreAbcdefg3565 | tar | chemotaxis protein | 3.159 | 1.659366565 | up |
|  | GeFixPreAbcdefg2730 | wza | polysaccharide biosynthesis protein GumB | 2.575 | 1.364366896 | up |
| Sulfur metabolism | GeFixPreAbcdefg3419 | cysJ | sulfite reductase | 7.492 | 2.905435157 | up |
|  | GeFixPreAbcdefg1232 | cysP | sulfate transporter subunit | 3.397 | 1.764316453 | up |
|  | GeFixPreAbcdefg3418 | cysD | sulfate adenylyltransferase small subunit | 4.846 | 2.276864801 | up |
|  | GeFixPreAbcdefg3428 | cysK | cysteine synthase A | 3.172 | 1.665585872 | up |
|  | GeFixPreAbcdefg1233 | cysU | sulfate ABC transporter permease subunit CysT | 2.547 | 1.34886759 | up |
|  | GeFixPreAbcdefg1065 | ssuD | alkanesulfonate monooxygenase | 3.597 | 1.846863535 | up |
|  | GeFixPreAbcdefg1066 | ssuE | FMN reductase | 10.356 | 3.372464113 | up |
|  | GeFixPreAbcdefg0407 | ssuE | FMN reductase | 0.187 | -2.420407998 | down |
|  | GeFixPreAbcdefg3175 | metB | cystathionine gamma-synthase | 3.461 | 1.791147548 | up |
|  | GeFixPreAbcdefg3176 | metX | homoserine acetyltransferase | 3.159 | 1.659472819 | up |
|  | GeFixPreAbcdefg1044 | tauD | taurine dioxygenase | 16.394 | 4.035127195 | up |
| Histidine metabolism | GeFixPreAbcdefg1878 | hutU | urocanate hydratase | 4.197 | 2.069307453 | up |
|  | GeFixPreAbcdefg1882 | - | formimidoylglutamate deiminase | 4.64 | 2.2141186 | up |
|  | GeFixPreAbcdefg1880 | hutH | histidine ammonia-lyase | 4.639 | 2.21370405 | up |
|  | GeFixPreAbcdefg1881 | hutI | imidazolonepropionase | 4.2 | 2.070272255 | up |
|  | GeFixPreAbcdefg3530 | hisC | aminotransferase | 2.176 | 1.121502033 | up |
|  | GeFixPreAbcdefg1879 | hutG | N-formylglutamate deformylase | 3.584 | 1.84142827 | up |
|  | GeFixPreAbcdefg2075 | hisB | bifunctional imidazole glycerol-phosphate dehydratase/histidinol phosphatase | 0.378 | -1.404491077 | down |
|  | GeFixPreAbcdefg2079 | hisIE | bifunctional phosphoribosyl-AMP cyclohydrolase/phosphoribosyl-ATP diphosphatase | 0.299 | -1.741950233 | down |
|  | GeFixPreAbcdefg2077 | hisA | 1-(5-phosphoribosyl)-5-[(5-phosphoribosylamino)methylideneamino]imidazole-4-carboxamide isomerase | 0.393 | -1.34656043 | down |
|  | GeFixPreAbcdefg2074 | hisC | histidinol-phosphate transaminase | 0.444 | -1.172048288 | down |
|  | GeFixPreAbcdefg2078 | hisF | imidazole glycerol phosphate synthase subunit HisF | 0.264 | -1.923641075 | down |
|  | GeFixPreAbcdefg2076 | hisH | imidazole glycerol phosphate synthase, glutamine amidotransferase subunit | 0.311 | -1.684801257 | down |
| Oxidative phosphorylate | GeFixPreAbcdefg2803 | nuoM | NADH-quinone oxidoreductase subunit M | 0.17 | -2.558172855 | down |
|  | GeFixPreAbcdefg2804 | nuoL | NADH-quinone oxidoreductase subunit L | 0.198 | -2.333009539 | down |
|  | GeFixPreAbcdefg2802 | nuoN | NADH:ubiquinone oxidoreductase subunit N | 0.201 | -2.314119719 | down |
|  | GeFixPreAbcdefg2807 | nuoI | NADH-quinone oxidoreductase subunit I | 0.325 | -1.619912367 | down |
|  | GeFixPreAbcdefg2808 | nuoH | NADH-quinone oxidoreductase subunit H | 0.271 | -1.885971658 | down |
|  | GeFixPreAbcdefg2806 | nuoJ | NADH:ubiquinone oxidoreductase subunit J | 0.265 | -1.915790763 | down |
|  | GeFixPreAbcdefg2805 | nuoK | NADH-quinone oxidoreductase subunit K | 0.221 | -2.17585818 | down |
|  | GeFixPreAbcdefg2810 | nuoF | NADH oxidoreductase (quinone) subunit F | 0.461 | -1.118203858 | down |
|  | GeFixPreAbcdefg2809 | nuoG | NADH dehydrogenase | 0.354 | -1.498016714 | down |
|  | GeFixPreAbcdefg1483 | cyoB | cytochrome o ubiquinol oxidase subunit I | 0.41 | -1.287381927 | down |
|  | GeFixPreAbcdefg1484 | cyoC | cytochrome o ubiquinol oxidase subunit III | 0.272 | -1.877428075 | down |
|  | GeFixPreAbcdefg2487 | cydA | cytochrome d ubiquinol oxidase, subunit II | 0.056 | -4.146212098 | down |
|  | GeFixPreAbcdefg3110 | cydB | cytochrome d ubiquinol oxidase subunit II | 0.415 | -1.26931684 | down |
|  | GeFixPreAbcdefg2488 | cydB | cytochrome d ubiquinol oxidase subunit II | 0.094 | -3.40692718 | down |
|  | GeFixPreAbcdefg3844 | cydB | cytochrome d ubiquinol oxidase subunit II | 2.268 | 1.181701735 | up |
|  | GeFixPreAbcdefg2489 | - | putative membrane protein | 0.093 | -3.425098866 | down |

**Table S2 Expression levels of xanthan gum synthetic genes during the fermentation process**

| **Gene_id** | **Gene_name** | **Gene_description** | **GC1_6** | **GC1_4** | **GC1_2** | **GC2_6** | **GC2_4** | **GC2_2** |
| --- | --- | --- | --- | --- | --- | --- | --- | --- |
| GeFixPreAbcdefg2719 | gumM | polysaccharide biosynthesis protein GumM | 52.48 | 54.93 | 175.15 | 101.4 | 49.28 | 134.76 |
| GeFixPreAbcdefg2720 | gumL | polysaccharide biosynthesis protein GumL | 80.82 | 113.07 | 351.52 | 159.32 | 66.32 | 207.95 |
| GeFixPreAbcdefg2721 | gumK | glycosyl transferase family 1 | 62.55 | 82.71 | 201.33 | 131.34 | 37.7 | 151.72 |
| GeFixPreAbcdefg2722 | gumJ | lipopolysaccharide biosynthesis protein | 14.78 | 11.43 | 20.08 | 17.9 | 10.33 | 15.36 |
| GeFixPreAbcdefg2723 | gumI | GDP-mannose--glycolipid 4-beta-D-mannosyltransferase | 25.08 | 15.59 | 27.28 | 19.5 | 22.67 | 18.82 |
| GeFixPreAbcdefg2724 | gumH | glycosyl transferase family 1 | 19.26 | 17.16 | 24.94 | 16.62 | 15.38 | 14.34 |
| GeFixPreAbcdefg2725 | gumG | polysaccharide biosynthesis protein GumG | 20.1 | 13.24 | 17.14 | 20.28 | 21.36 | 15.85 |
| GeFixPreAbcdefg2726 | gumF | polysaccharide biosynthesis protein GumF | 25.66 | 20.44 | 24.04 | 20.71 | 32.44 | 19.53 |
| GeFixPreAbcdefg2727 | gumE | polysaccharide biosynthesis protein GumE | 67.65 | 64.76 | 61.91 | 66.99 | 131.71 | 58.24 |
| GeFixPreAbcdefg2728 | gumD | undecaprenyl-phosphate glucose phosphotransferase | 675.07 | 800.86 | 522.87 | 918.93 | 1126.65 | 552.93 |
| GeFixPreAbcdefg2729 | gumC | polysaccharide biosynthesis protein GumC | 330.83 | 238.93 | 145.88 | 116.9 | 185.24 | 117.28 |
| GeFixPreAbcdefg2730 | gumB | polysaccharide biosynthesis protein GumB | 340.4 | 392.39 | 222.82 | 200.18 | 172.45 | 103.82 |

**Table S3 The expression of DEGs in sulfur metabolism pathway of GC1_6d vs 4d and GC2_4d vs 2d**

| **Gene_id** | **Gene ame** | **Gene description** | **FC(G1_6/G1_4)** | **Log2FC(G1_6/G1_4)** | **Padjust** | **Regulate** |
| --- | --- | --- | --- | --- | --- | --- |
| GeFixPreAbcdefg3176 | metX | homoserine acetyltransferase | 2.487 | 1.314683 | 1.05E-07 | up |
| GeFixPreAbcdefg3175 | metB | cystathionine gamma-synthase | 3.844 | 1.942559 | 6.79E-05 | up |
| GeFixPreAbcdefg1065 | ssuD | alkanesulfonate monooxygenase | 0.458 | -1.12659 | 6.79E-05 | down |
| GeFixPreAbcdefg1064 | ssuA | ABC transporter substrate-binding protein | 0.46 | -1.11928 | 0.000742 | down |
| GeFixPreAbcdefg1044 | tauD | taurine dioxygenase | 0.405 | -1.30411 | 0.004054 | down |
| **Gene_id** | **Gene ame** | **Gene description** | **FC(G2_4/G2_2)** | **Log2FC(G2_4/G2_2)** | **Padjust** | **Regulate** |
| GeFixPreAbcdefg3418 | cysD | sulfate adenylyltransferase small subunit | 3.544 | 1.825261571 | 3.91E-41 | up |
| GeFixPreAbcdefg3421 | cysH | phosphoadenosine phosphosulfate reductase | 2.963 | 1.566962027 | 1.07E-33 | up |
| GeFixPreAbcdefg3420 | cysI | sulfite reductase subunit beta | 2.449 | 1.292083375 | 7.82E-22 | up |
| GeFixPreAbcdefg3419 | cysJ | sulfite reductase | 3.487 | 1.801904793 | 9.76E-36 | up |
| GeFixPreAbcdefg3428 | cysK | cysteine synthase A | 4.55 | 2.185811463 | 3.52E-45 | up |
| GeFixPreAbcdefg3417 | cysNC | adenylyl-sulfate kinase | 3.483 | 1.800195991 | 7.24E-40 | up |
| GeFixPreAbcdefg3175 | metB | cystathionine gamma-synthase | 12.203 | 3.609179876 | 1.59E-09 | up |
| GeFixPreAbcdefg2483 | metX | homoserine O-acetyltransferase | 0.454 | -1.140324863 | 1.09E-11 | down |
| GeFixPreAbcdefg1831 | sseA | sulfurtransferase | 2.992 | 1.580959147 | 1.01E-21 | up |
| GeFixPreAbcdefg1064 | ssuA | ABC transporter substrate-binding protein | 3.446 | 1.785075756 | 2.74E-26 | up |
| GeFixPreAbcdefg1062 | ssuB | sulfonate ABC transporter ATP-binding protein | 2.351 | 1.233224371 | 0.000162512 | up |
| GeFixPreAbcdefg1065 | ssuD | alkanesulfonate monooxygenase | 3.534 | 1.821146013 | 7.00E-18 | up |
| GeFixPreAbcdefg0407 | ssuE | FMN reductase | 3.347 | 1.742935757 | 1.35E-48 | up |
| GeFixPreAbcdefg1066 | ssuE | FMN reductase | 2.41 | 1.26887041 | 5.32E-06 | up |
| GeFixPreAbcdefg1044 | tauD | taurine dioxygenase | 7.244 | 2.856721565 | 3.06E-74 | up |

**Table S4 The expression of DEGs in KEGG enrichment pathways of GC2_6d vs 4d**

| **Pathway** | **ID** | **Gene name** | **Gene description** | **FC(G2_6/G2_4)** | **Log2FC(G2_6/G2_4)** | **Regulate** |
| --- | --- | --- | --- | --- | --- | --- |
| Bacterial chemotaxis | GeFixPreAbcdefg2144 | - | chemotaxis protein | 18.516 | 4.210669536 | up |
|  | GeFixPreAbcdefg2145 | - | chemotaxis protein | 7.661 | 2.937599643 | up |
|  | GeFixPreAbcdefg1527 | - | chemotaxis protein | 7.212 | 2.850445684 | up |
|  | GeFixPreAbcdefg2140 | - | chemotaxis protein | 4.352 | 2.121737057 | up |
|  | GeFixPreAbcdefg2153 | - | chemotaxis protein | 5.384 | 2.428796584 | up |
|  | GeFixPreAbcdefg2189 | cheA | chemotaxis protein CheA | 24.715 | 4.627295458 | up |
|  | GeFixPreAbcdefg2154 | cheA | chemotaxis protein CheA | 14.167 | 3.824447388 | up |
|  | GeFixPreAbcdefg2977 | cheA | chemotaxis protein CheA | 6.833 | 2.772561496 | up |
|  | GeFixPreAbcdefg2130 | cheB | chemotaxis response regulator protein-glutamate methylesterase | 6.059 | 2.599091688 | up |
|  | GeFixPreAbcdefg2982 | cheB | chemotaxis response regulator protein-glutamate methylesterase | 3.858 | 1.94769957 | up |
|  | GeFixPreAbcdefg2131 | cheD | chemoreceptor glutamine deamidase CheD | 4.694 | 2.230938631 | up |
|  | GeFixPreAbcdefg2132 | cheR | chemotaxis protein CheR | 39.151 | 5.290976493 | up |
|  | GeFixPreAbcdefg2981 | cheR | SAM-dependent methyltransferase | 17.299 | 4.112598944 | up |
|  | GeFixPreAbcdefg2254 | cheV | conserved hypothetical protein | 20.25 | 4.339840426 | up |
|  | GeFixPreAbcdefg2157 | cheW | chemotaxis protein | 26.173 | 4.70999448 | up |
|  | GeFixPreAbcdefg2135 | cheW | chemotaxis protein CheW | 21.774 | 4.444517818 | up |
|  | GeFixPreAbcdefg2572 | cheW | hypothetical protein | 16.166 | 4.014892762 | up |
|  | GeFixPreAbcdefg2979 | cheW | hypothetical protein | 12.75 | 3.672449287 | up |
|  | GeFixPreAbcdefg2191 | cheY | response regulator | 31.87 | 4.994140713 | up |
|  | GeFixPreAbcdefg2155 | cheY | Fis family transcriptional regulator | 17.956 | 4.166426662 | up |
|  | GeFixPreAbcdefg2190 | cheZ | chemotaxis protein | 39.088 | 5.288660723 | up |
|  | GeFixPreAbcdefg2219 | fliG | flagellar motor switch protein FliG | 12.802 | 3.678261346 | up |
|  | GeFixPreAbcdefg2212 | fliM | flagellar motor switch protein FliM | 27.61 | 4.787094677 | up |
|  | GeFixPreAbcdefg2211 | fliNY | flagellar motor switch protein FliN | 20.211 | 4.337082102 | up |
|  | GeFixPreAbcdefg2263 | mcp | methyl-accepting chemotaxis (MCP) signaling domain protein | 22.854 | 4.51438655 | up |
|  | GeFixPreAbcdefg2133 | mcp | chemotaxis protein | 18.856 | 4.236922572 | up |
|  | GeFixPreAbcdefg2137 | mcp | chemotaxis protein | 26.781 | 4.743120933 | up |
|  | GeFixPreAbcdefg2573 | mcp | chemotaxis protein | 14.8 | 3.887556838 | up |
|  | GeFixPreAbcdefg2978 | mcp | chemotaxis protein | 16.421 | 4.037508689 | up |
|  | GeFixPreAbcdefg2148 | mcp | chemotaxis protein | 16.631 | 4.055770808 | up |
|  | GeFixPreAbcdefg2147 | mcp | chemotaxis protein | 8.577 | 3.100458627 | up |
|  | GeFixPreAbcdefg2143 | mcp | chemotaxis protein | 4.762 | 2.251616592 | up |
|  | GeFixPreAbcdefg2434 | mcp | HAMP domain-containing protein | 2.871 | 1.521775279 | up |
|  | GeFixPreAbcdefg3354 | mcp | chemotaxis protein | 2.892 | 1.532275304 | up |
|  | GeFixPreAbcdefg2160 | motA | flagellar motor protein | 7.894 | 2.980835796 | up |
|  | GeFixPreAbcdefg3798 | motA | flagellar motor protein MotA | 2.77 | 1.470014817 | up |
|  | GeFixPreAbcdefg2159 | motB | flagellar motor protein MotD | 44.582 | 5.47839603 | up |
|  | GeFixPreAbcdefg3799 | motB | flagellar motor protein MotB | 4.224 | 2.078690264 | up |
| flagellar assembly | GeFixPreAbcdefg2255 | flgA | flagellar basal body P-ring biosynthesis protein FlgA | 74.225 | 6.21382456 | up |
|  | GeFixPreAbcdefg2253 | flgB | flagellar biosynthesis protein FlgB | 58.844 | 5.878831684 | up |
|  | GeFixPreAbcdefg2252 | flgC | flagellar basal body rod protein FlgC | 46.818 | 5.548996225 | up |
|  | GeFixPreAbcdefg2251 | flgD | flagellar basal body rod modification protein | 30.688 | 4.939604238 | up |
|  | GeFixPreAbcdefg2250 | flgE | flagellar hook protein FlgE | 37.051 | 5.211433046 | up |
|  | GeFixPreAbcdefg2249 | flgF | flagellar basal body rod protein FlgF | 30.87 | 4.948144935 | up |
|  | GeFixPreAbcdefg2247 | flgG | flagellar basal body rod protein FlgG | 72.678 | 6.183444917 | up |
|  | GeFixPreAbcdefg2246 | flgH | flagellar basal body L-ring protein | 48.371 | 5.596083069 | up |
|  | GeFixPreAbcdefg2245 | flgI | flagellar P-ring protein | 36.946 | 5.207336195 | up |
|  | GeFixPreAbcdefg2243 | flgK | flagellar hook protein FlgK | 27.585 | 4.78580028 | up |
|  | GeFixPreAbcdefg2242 | flgL | flagellar hook protein FlgL | 14.382 | 3.84614233 | up |
|  | GeFixPreAbcdefg2256 | flgM | flagellar biosynthesis anti-sigma factor FlgM | 29.923 | 4.903203017 | up |
|  | GeFixPreAbcdefg2201 | flhA | flagellar biosynthesis protein FlhA | 30.56 | 4.933564808 | up |
|  | GeFixPreAbcdefg2202 | flhB | flagellar biosynthesis protein FlhB | 38.42 | 5.263775784 | up |
|  | GeFixPreAbcdefg2192 | fliA | RNA polymerase sigma factor FliA | 59.134 | 5.885919187 | up |
|  | GeFixPreAbcdefg2241 | fliC | flagellin | 36.814 | 5.202194358 | up |
|  | GeFixPreAbcdefg2240 | fliD | flagellar protein | 36.453 | 5.187980009 | up |
|  | GeFixPreAbcdefg2221 | fliE | flagellar hook-basal body protein | 61.701 | 5.947226895 | up |
|  | GeFixPreAbcdefg2220 | fliF | flagellar M-ring protein FliF | 37.231 | 5.218421582 | up |
|  | GeFixPreAbcdefg2219 | fliG | flagellar motor switch protein FliG | 12.802 | 3.678261346 | up |
|  | GeFixPreAbcdefg2218 | fliH | flagellar assembly protein FliH | 8.83 | 3.142472207 | up |
|  | GeFixPreAbcdefg2217 | fliI | flagellar protein export ATPase FliI | 7.297 | 2.867354035 | up |
|  | GeFixPreAbcdefg0256 | fliI | ATP synthase | 2.217 | 1.148310012 | up |
|  | GeFixPreAbcdefg2216 | fliJ | flagellar export protein FliJ | 7.133 | 2.834549451 | up |
|  | GeFixPreAbcdefg2215 | fliK | flagellar protein | 5.456 | 2.447902087 | up |
|  | GeFixPreAbcdefg2212 | fliM | flagellar motor switch protein FliM | 27.61 | 4.787094677 | up |
|  | GeFixPreAbcdefg2211 | fliNY | flagellar motor switch protein FliN | 20.211 | 4.337082102 | up |
|  | GeFixPreAbcdefg2210 | fliOZ | flagellar biosynthetic protein FliO | 28.098 | 4.812383914 | up |
|  | GeFixPreAbcdefg2209 | fliP | flagellar biosynthetic protein FliP | 16.79 | 4.069507407 | up |
|  | GeFixPreAbcdefg2208 | fliQ | flagellar biosynthesis | 12.462 | 3.639481881 | up |
|  | GeFixPreAbcdefg2207 | fliR | flagellar biosynthetic protein FliR | 20.369 | 4.348291709 | up |
|  | GeFixPreAbcdefg2239 | fliS | flagellar protein FliS | 19.169 | 4.260704804 | up |
|  | GeFixPreAbcdefg2160 | motA | flagellar motor protein | 7.894 | 2.980835796 | up |
|  | GeFixPreAbcdefg3798 | motA | flagellar motor protein MotA | 2.77 | 1.470014817 | up |
|  | GeFixPreAbcdefg2159 | motB | flagellar motor protein MotD | 44.582 | 5.47839603 | up |
|  | GeFixPreAbcdefg3799 | motB | flagellar motor protein MotB | 4.224 | 2.078690264 | up |
| Two-component system | GeFixPreAbcdefg2144 | - | chemotaxis protein | 18.516 | 4.210669536 | up |
|  | GeFixPreAbcdefg0030 | - | cellulase | 49.593 | 5.632070112 | up |
|  | GeFixPreAbcdefg2145 | - | chemotaxis protein | 7.661 | 2.937599643 | up |
|  | GeFixPreAbcdefg1527 | - | chemotaxis protein | 7.212 | 2.850445684 | up |
|  | GeFixPreAbcdefg2489 | - | putative membrane protein | 3.054 | 1.610628709 | up |
|  | GeFixPreAbcdefg2140 | - | chemotaxis protein | 4.352 | 2.121737057 | up |
|  | GeFixPreAbcdefg0778 | - | endoglucanase | 0.473 | -1.080587773 | down |
|  | GeFixPreAbcdefg2153 | - | chemotaxis protein | 5.384 | 2.428796584 | up |
|  | GeFixPreAbcdefg0787 | algR | DNA-binding response regulator | 2.446 | 1.290560606 | up |
|  | GeFixPreAbcdefg3403 | cbhA | 1,4-beta-cellobiosidase | 2.84 | 1.505789226 | up |
|  | GeFixPreAbcdefg2189 | cheA | chemotaxis protein CheA | 24.715 | 4.627295458 | up |
|  | GeFixPreAbcdefg2154 | cheA | chemotaxis protein CheA | 14.167 | 3.824447388 | up |
|  | GeFixPreAbcdefg2977 | cheA | chemotaxis protein CheA | 6.833 | 2.772561496 | up |
|  | GeFixPreAbcdefg2130 | cheB | chemotaxis response regulator protein-glutamate methylesterase | 6.059 | 2.599091688 | up |
|  | GeFixPreAbcdefg2982 | cheB | chemotaxis response regulator protein-glutamate methylesterase | 3.858 | 1.94769957 | up |
|  | GeFixPreAbcdefg2132 | cheR | chemotaxis protein CheR | 39.151 | 5.290976493 | up |
|  | GeFixPreAbcdefg2981 | cheR | SAM-dependent methyltransferase | 17.299 | 4.112598944 | up |
|  | GeFixPreAbcdefg2254 | cheV | conserved hypothetical protein | 20.25 | 4.339840426 | up |
|  | GeFixPreAbcdefg2157 | cheW | chemotaxis protein | 26.173 | 4.70999448 | up |
|  | GeFixPreAbcdefg2135 | cheW | chemotaxis protein CheW | 21.774 | 4.444517818 | up |
|  | GeFixPreAbcdefg2572 | cheW | hypothetical protein | 16.166 | 4.014892762 | up |
|  | GeFixPreAbcdefg2979 | cheW | hypothetical protein | 12.75 | 3.672449287 | up |
|  | GeFixPreAbcdefg2191 | cheY | response regulator | 31.87 | 4.994140713 | up |
|  | GeFixPreAbcdefg2155 | cheY | Fis family transcriptional regulator | 17.956 | 4.166426662 | up |
|  | GeFixPreAbcdefg3238 | chpB | chemotaxis protein | 5.051 | 2.33663408 | up |
|  | GeFixPreAbcdefg3237 | chpC | chemotaxis protein CheW | 4.287 | 2.099878655 | up |
|  | GeFixPreAbcdefg3843 | cydA | cytochrome D ubiquinol oxidase subunit I | 2.298 | 1.200370382 | up |
|  | GeFixPreAbcdefg2487 | cydA | cytochrome d ubiquinol oxidase, subunit II | 2.153 | 1.106541417 | up |
|  | GeFixPreAbcdefg2488 | cydB | cytochrome d ubiquinol oxidase subunit II | 2.574 | 1.363812165 | up |
|  | GeFixPreAbcdefg3597 | dctA | C4-dicarboxylate transporter | 0.322 | -1.634811944 | down |
|  | GeFixPreAbcdefg2256 | flgM | flagellar biosynthesis anti-sigma factor FlgM | 29.923 | 4.903203017 | up |
|  | GeFixPreAbcdefg2192 | fliA | RNA polymerase sigma factor FliA | 59.134 | 5.885919187 | up |
|  | GeFixPreAbcdefg2241 | fliC | flagellin | 36.814 | 5.202194358 | up |
|  | GeFixPreAbcdefg2605 | glnA | glutamine synthetase | 2.012 | 1.00866672 | up |
|  | GeFixPreAbcdefg1677 | glnD | bifunctional uridylyltransferase/uridylyl-removing protein | 0.286 | -1.808380368 | down |
|  | GeFixPreAbcdefg2263 | mcp | methyl-accepting chemotaxis (MCP) signaling domain protein | 22.854 | 4.51438655 | up |
|  | GeFixPreAbcdefg2133 | mcp | chemotaxis protein | 18.856 | 4.236922572 | up |
|  | GeFixPreAbcdefg2137 | mcp | chemotaxis protein | 26.781 | 4.743120933 | up |
|  | GeFixPreAbcdefg2573 | mcp | chemotaxis protein | 14.8 | 3.887556838 | up |
|  | GeFixPreAbcdefg2978 | mcp | chemotaxis protein | 16.421 | 4.037508689 | up |
|  | GeFixPreAbcdefg2148 | mcp | chemotaxis protein | 16.631 | 4.055770808 | up |
|  | GeFixPreAbcdefg2147 | mcp | chemotaxis protein | 8.577 | 3.100458627 | up |
|  | GeFixPreAbcdefg2143 | mcp | chemotaxis protein | 4.762 | 2.251616592 | up |
|  | GeFixPreAbcdefg2434 | mcp | HAMP domain-containing protein | 2.871 | 1.521775279 | up |
|  | GeFixPreAbcdefg3354 | mcp | chemotaxis protein | 2.892 | 1.532275304 | up |
|  | GeFixPreAbcdefg3974 | mdtA | efflux transporter periplasmic adaptor subunit | 0.176 | -2.504582935 | down |
|  | GeFixPreAbcdefg3975 | mdtB | acriflavine resistance protein B | 0.137 | -2.866661847 | down |
|  | GeFixPreAbcdefg3977 | mdtC | acriflavin resistance protein | 0.224 | -2.159433998 | down |
|  | GeFixPreAbcdefg2160 | motA | flagellar motor protein | 7.894 | 2.980835796 | up |
|  | GeFixPreAbcdefg3798 | motA | flagellar motor protein MotA | 2.77 | 1.470014817 | up |
|  | GeFixPreAbcdefg1256 | phoB | DNA-binding response regulator | 0.371 | -1.429229127 | down |
|  | GeFixPreAbcdefg1255 | phoR | histidine kinase | 0.373 | -1.422335385 | down |
|  | GeFixPreAbcdefg1818 | pstS | phosphate ABC transporter substrate-binding protein PstS | 0.147 | -2.76268771 | down |
|  | GeFixPreAbcdefg3371 | pilA | competence protein | 3.174 | 1.666252839 | up |
|  | GeFixPreAbcdefg3244 | pilG | pilus protein | 2.378 | 1.249567957 | up |
|  | GeFixPreAbcdefg3243 | pilH | two-component system response regulator protein | 2.942 | 1.556622986 | up |
|  | GeFixPreAbcdefg1361 | pilH | response regulator | 2.156 | 1.108416542 | up |
|  | GeFixPreAbcdefg3242 | pilI | pilus biogenesis protein | 2.491 | 1.316742923 | up |
|  | GeFixPreAbcdefg2118 | rpfC | hybrid sensor histidine kinase/response regulator | 2.692 | 1.428745589 | up |
|  | GeFixPreAbcdefg2116 | rpfG | two-component system response regulator | 5.434 | 2.441956863 | up |
|  | GeFixPreAbcdefg3099 | rpoN | RNA polymerase factor sigma-54 | 0.391 | -1.355049188 | down |
|  | GeFixPreAbcdefg2235 | rpoN | RNA polymerase sigma-54 factor | 2.861 | 1.516503684 | up |
| Sulfur metabolism | GeFixPreAbcdefg3418 | cysD | sulfate adenylyltransferase small subunit | 0.117 | -3.09420437 | down |
|  | GeFixPreAbcdefg3421 | cysH | phosphoadenosine phosphosulfate reductase | 0.097 | -3.365687408 | down |
|  | GeFixPreAbcdefg3420 | cysI | sulfite reductase subunit beta | 0.097 | -3.372832928 | down |
|  | GeFixPreAbcdefg3419 | cysJ | sulfite reductase | 0.039 | -4.66224896 | down |
|  | GeFixPreAbcdefg4165 | cysJ | iron-uptake factor | 0.2 | -2.325130529 | down |
|  | GeFixPreAbcdefg3075 | cysJ | sulfite reductase | 0.448 | -1.157201197 | down |
|  | GeFixPreAbcdefg3428 | cysK | cysteine synthase A | 0.16 | -2.642910646 | down |
|  | GeFixPreAbcdefg3730 | cysK | cysteine synthase | 0.465 | -1.105450522 | down |
|  | GeFixPreAbcdefg3417 | cysNC | adenylyl-sulfate kinase | 0.101 | -3.300642472 | down |
|  | GeFixPreAbcdefg1233 | cysU | sulfate ABC transporter permease subunit CysT | 0.473 | -1.078958098 | down |
|  | GeFixPreAbcdefg1831 | sseA | sulfurtransferase | 0.407 | -1.298564014 | down |
|  | GeFixPreAbcdefg1064 | ssuA | ABC transporter substrate-binding protein | 0.093 | -3.428621095 | down |
|  | GeFixPreAbcdefg1062 | ssuB | sulfonate ABC transporter ATP-binding protein | 0.119 | -3.073286153 | down |
|  | GeFixPreAbcdefg1063 | ssuC | ABC transporter permease | 0.102 | -3.288837984 | down |
|  | GeFixPreAbcdefg1065 | ssuD | alkanesulfonate monooxygenase | 0.137 | -2.866112224 | down |
|  | GeFixPreAbcdefg1066 | ssuE | FMN reductase | 0.204 | -2.296139055 | down |
|  | GeFixPreAbcdefg1044 | tauD | taurine dioxygenase | 0.046 | -4.430125609 | down |
| Starch and sucrose metabolism | GeFixPreAbcdefg0030 | - | cellulase | 49.593 | 5.632070112 | up |
|  | GeFixPreAbcdefg0778 | - | endoglucanase | 0.473 | -1.080587773 | down |
|  | GeFixPreAbcdefg1010 | amyA | alpha-amylase | 5.149 | 2.364341516 | up |
|  | GeFixPreAbcdefg0931 | bcsA | cellulose synthase catalytic subunit (UDP-forming) | 2.073 | 1.051469633 | up |
|  | GeFixPreAbcdefg1697 | bglX | glycosyl hydrolase | 3.292 | 1.718990553 | up |
|  | GeFixPreAbcdefg3997 | bglX | beta-glucosidase | 2.975 | 1.57301695 | up |
|  | GeFixPreAbcdefg2030 | bglX | 1,4-beta-D-glucan glucohydrolase | 0.457 | -1.128206267 | down |
|  | GeFixPreAbcdefg3215 | bglX | beta-glucosidase | 2.082 | 1.058138595 | up |
|  | GeFixPreAbcdefg4380 | bglX | glucan 1,4-alpha-glucosidase | 2.081 | 1.057073777 | up |
|  | GeFixPreAbcdefg3403 | cbhA | 1,4-beta-cellobiosidase | 2.84 | 1.505789226 | up |
|  | GeFixPreAbcdefg0492 | glgA | starch synthase | 5.46 | 2.448890892 | up |
|  | GeFixPreAbcdefg0493 | glgB | 1,4-alpha-glucan branching enzyme | 4.975 | 2.314665388 | up |
|  | GeFixPreAbcdefg0148 | glgE | alpha-amylase | 2.518 | 1.332518585 | up |
|  | GeFixPreAbcdefg0495 | malQ | 4-alpha-glucanotransferase | 4.451 | 2.154279597 | up |
|  | GeFixPreAbcdefg2751 | malZ | alpha-glucosidase | 3.515 | 1.813560243 | up |
|  | GeFixPreAbcdefg2748 | malZ | alpha-glucosidase | 2.784 | 1.477277837 | up |
|  | GeFixPreAbcdefg1798 | scrK | fructokinase | 0.185 | -2.437148479 | down |
|  | GeFixPreAbcdefg0149 | treS | trehalose synthase | 2.066 | 1.046637449 | up |
|  | GeFixPreAbcdefg3386 | treX | glycogen debranching enzyme | 2.777 | 1.473413728 | up |
|  | GeFixPreAbcdefg0498 | treX | glycogen debranching enzyme | 0.471 | -1.087123044 | down |
|  | GeFixPreAbcdefg0496 | treY | malto-oligosyltrehalose synthase | 3.925 | 1.972721286 | up |
|  | GeFixPreAbcdefg0494 | treZ | malto-oligosyltrehalose trehalohydrolase | 4.271 | 2.094692157 | up |

**Table S5 The expression of DEGs in KEGG enrichment pathways of 4d_GC2 vs GC1**

| **Pathway** | **Gene_id** | **Gene name** | **Gene description** | **FC(GC2_4/GC1_4)** | **Log2FC(GC2_4/GC1_4)** | **Regulate** |
| --- | --- | --- | --- | --- | --- | --- |
| Bacterial chemotaxis | GeFixPreAbcdefg2159 | motB | flagellar motor protein MotD | 0.006 | -7.40124 | down |
|  | GeFixPreAbcdefg2157 | cheW | chemotaxis protein | 0.006 | -7.29094 | down |
|  | GeFixPreAbcdefg2155 | cheY | Fis family transcriptional regulator | 0.011 | -6.48697 | down |
|  | GeFixPreAbcdefg2154 | cheA | chemotaxis protein CheA | 0.011 | -6.44457 | down |
|  | GeFixPreAbcdefg2153 | - | chemotaxis protein | 0.043 | -4.55449 | down |
|  | GeFixPreAbcdefg2151 | - | chemotaxis protein | 0.133 | -2.91305 | down |
|  | GeFixPreAbcdefg1527 | - | chemotaxis protein | 0.009 | -6.76891 | down |
|  | GeFixPreAbcdefg2979 | cheW | hypothetical protein | 0.014 | -6.18802 | down |
|  | GeFixPreAbcdefg2148 | mcp | chemotaxis protein | 0.008 | -6.95739 | down |
|  | GeFixPreAbcdefg2144 | - | chemotaxis protein | 0.011 | -6.5109 | down |
|  | GeFixPreAbcdefg2145 | - | chemotaxis protein | 0.037 | -4.74468 | down |
|  | GeFixPreAbcdefg2147 | mcp | chemotaxis protein | 0.027 | -5.19152 | down |
|  | GeFixPreAbcdefg2140 | - | chemotaxis protein | 0.06 | -4.04796 | down |
|  | GeFixPreAbcdefg2143 | mcp | chemotaxis protein | 0.038 | -4.7326 | down |
|  | GeFixPreAbcdefg2254 | cheV | conserved hypothetical protein | 0.006 | -7.3405 | down |
|  | GeFixPreAbcdefg2981 | cheR | SAM-dependent methyltransferase | 0.016 | -5.97912 | down |
|  | GeFixPreAbcdefg2982 | cheB | chemotaxis response regulator protein-glutamate methylesterase | 0.107 | -3.23028 | down |
|  | GeFixPreAbcdefg3798 | motA | flagellar motor protein MotA | 0.077 | -3.70287 | down |
|  | GeFixPreAbcdefg3799 | motB | flagellar motor protein MotB | 0.051 | -4.29802 | down |
|  | GeFixPreAbcdefg2263 | mcp | methyl-accepting chemotaxis (MCP) signaling domain protein | 0.008 | -6.94372 | down |
|  | GeFixPreAbcdefg2573 | mcp | chemotaxis protein | 0.011 | -6.4745 | down |
|  | GeFixPreAbcdefg2572 | cheW | hypothetical protein | 0.013 | -6.29528 | down |
|  | GeFixPreAbcdefg2160 | motA | flagellar motor protein | 0.018 | -5.77568 | down |
|  | GeFixPreAbcdefg3565 | tar | chemotaxis protein | 0.305 | -1.71318 | down |
|  | GeFixPreAbcdefg2191 | cheY | response regulator | 0.005 | -7.57462 | down |
|  | GeFixPreAbcdefg2190 | cheZ | chemotaxis protein | 0.005 | -7.62109 | down |
|  | GeFixPreAbcdefg2219 | fliG | flagellar motor switch protein FliG | 0.016 | -5.94765 | down |
|  | GeFixPreAbcdefg2211 | fliNY | flagellar motor switch protein FliN | 0.013 | -6.27115 | down |
|  | GeFixPreAbcdefg2212 | fliM | flagellar motor switch protein FliM | 0.012 | -6.33489 | down |
|  | GeFixPreAbcdefg2189 | cheA | chemotaxis protein CheA | 0.009 | -6.81891 | down |
|  | GeFixPreAbcdefg2135 | cheW | chemotaxis protein CheW | 0.006 | -7.41744 | down |
|  | GeFixPreAbcdefg2978 | mcp | chemotaxis protein | 0.008 | -6.98104 | down |
|  | GeFixPreAbcdefg2137 | mcp | chemotaxis protein | 0.005 | -7.60302 | down |
|  | GeFixPreAbcdefg1981 | mcp | chemotaxis protein | 0.2 | -2.31959 | down |
|  | GeFixPreAbcdefg2133 | mcp | chemotaxis protein | 0.023 | -5.45358 | down |
|  | GeFixPreAbcdefg2132 | cheR | chemotaxis protein CheR | 0.004 | -7.95466 | down |
|  | GeFixPreAbcdefg2977 | cheA | chemotaxis protein CheA | 0.017 | -5.83962 | down |
|  | GeFixPreAbcdefg2434 | mcp | HAMP domain-containing protein | 0.293 | -1.77073 | down |
|  | GeFixPreAbcdefg3354 | mcp | chemotaxis protein | 0.053 | -4.24179 | down |
| Flagellar assembly | GeFixPreAbcdefg2239 | fliS | flagellar protein FliS | 0.011 | -6.569628103 | down |
|  | GeFixPreAbcdefg2159 | motB | flagellar motor protein MotD | 0.006 | -7.4012373 | down |
|  | GeFixPreAbcdefg2247 | flgG | flagellar basal body rod protein FlgG | 0.003 | -8.46915567 | down |
|  | GeFixPreAbcdefg2246 | flgH | flagellar basal body L-ring protein | 0.003 | -8.361201582 | down |
|  | GeFixPreAbcdefg2245 | flgI | flagellar P-ring protein | 0.004 | -8.010728787 | down |
|  | GeFixPreAbcdefg2243 | flgK | flagellar hook protein FlgK | 0.006 | -7.276275597 | down |
|  | GeFixPreAbcdefg2242 | flgL | flagellar hook protein FlgL | 0.011 | -6.536864604 | down |
|  | GeFixPreAbcdefg2241 | fliC | flagellin | 0.005 | -7.557504442 | down |
|  | GeFixPreAbcdefg2240 | fliD | flagellar protein | 0.008 | -7.040284824 | down |
|  | GeFixPreAbcdefg2249 | flgF | flagellar basal body rod protein FlgF | 0.004 | -7.948379978 | down |
|  | GeFixPreAbcdefg2255 | flgA | flagellar basal body P-ring biosynthesis protein FlgA | 0.005 | -7.549492018 | down |
|  | GeFixPreAbcdefg2256 | flgM | flagellar biosynthesis anti-sigma factor FlgM | 0.01 | -6.642274405 | down |
|  | GeFixPreAbcdefg2250 | flgE | flagellar hook protein FlgE | 0.004 | -8.013494699 | down |
|  | GeFixPreAbcdefg2251 | flgD | flagellar basal body rod modification protein | 0.007 | -7.216467376 | down |
|  | GeFixPreAbcdefg2252 | flgC | flagellar basal body rod protein FlgC | 0.007 | -7.188503935 | down |
|  | GeFixPreAbcdefg2253 | flgB | flagellar biosynthesis protein FlgB | 0.006 | -7.409473834 | down |
|  | GeFixPreAbcdefg3798 | motA | flagellar motor protein MotA | 0.077 | -3.702871603 | down |
|  | GeFixPreAbcdefg3799 | motB | flagellar motor protein MotB | 0.051 | -4.298024771 | down |
|  | GeFixPreAbcdefg2160 | motA | flagellar motor protein | 0.018 | -5.775680519 | down |
|  | GeFixPreAbcdefg2209 | fliP | flagellar biosynthetic protein FliP | 0.015 | -6.031910914 | down |
|  | GeFixPreAbcdefg2208 | fliQ | flagellar biosynthesis | 0.029 | -5.091928319 | down |
|  | GeFixPreAbcdefg2202 | flhB | flagellar biosynthesis protein FlhB | 0.011 | -6.504704709 | down |
|  | GeFixPreAbcdefg2201 | flhA | flagellar biosynthesis protein FlhA | 0.008 | -6.9049703 | down |
|  | GeFixPreAbcdefg2192 | fliA | RNA polymerase sigma factor FliA | 0.002 | -8.741610827 | down |
|  | GeFixPreAbcdefg2218 | fliH | flagellar assembly protein FliH | 0.032 | -4.97600986 | down |
|  | GeFixPreAbcdefg2219 | fliG | flagellar motor switch protein FliG | 0.016 | -5.947649274 | down |
|  | GeFixPreAbcdefg2210 | fliOZ | flagellar biosynthetic protein FliO | 0.012 | -6.402018287 | down |
|  | GeFixPreAbcdefg2211 | fliNY | flagellar motor switch protein FliN | 0.013 | -6.271145533 | down |
|  | GeFixPreAbcdefg2212 | fliM | flagellar motor switch protein FliM | 0.012 | -6.33489476 | down |
|  | GeFixPreAbcdefg2215 | fliK | flagellar protein | 0.043 | -4.531005405 | down |
|  | GeFixPreAbcdefg2216 | fliJ | flagellar export protein FliJ | 0.039 | -4.664175802 | down |
|  | GeFixPreAbcdefg2217 | fliI | flagellar protein export ATPase FliI | 0.031 | -4.994705653 | down |
|  | GeFixPreAbcdefg2207 | fliR | flagellar biosynthetic protein FliR | 0.024 | -5.404573802 | down |
|  | GeFixPreAbcdefg2221 | fliE | flagellar hook-basal body protein | 0.01 | -6.700738708 | down |
|  | GeFixPreAbcdefg2220 | fliF | flagellar M-ring protein FliF | 0.008 | -7.051490073 | down |
| Two-component syst | GeFixPreAbcdefg3977 | mdtC | acriflavin resistance protein | 5.017 | 2.326682624 | up |
|  | GeFixPreAbcdefg2489 | - | putative membrane protein | 2.4 | 1.263278427 | up |
|  | GeFixPreAbcdefg2488 | cydB | cytochrome d ubiquinol oxidase subunit II | 2.252 | 1.170971495 | up |
|  | GeFixPreAbcdefg2235 | rpoN | RNA polymerase sigma-54 factor | 0.211 | -2.242970031 | down |
|  | GeFixPreAbcdefg2157 | cheW | chemotaxis protein | 0.006 | -7.290941519 | down |
|  | GeFixPreAbcdefg2155 | cheY | Fis family transcriptional regulator | 0.011 | -6.486968009 | down |
|  | GeFixPreAbcdefg2154 | cheA | chemotaxis protein CheA | 0.011 | -6.444569761 | down |
|  | GeFixPreAbcdefg2153 | - | chemotaxis protein | 0.043 | -4.554489119 | down |
|  | GeFixPreAbcdefg2151 | - | chemotaxis protein | 0.133 | -2.913052658 | down |
|  | GeFixPreAbcdefg0031 | - | cellulase | 0.335 | -1.576486542 | down |
|  | GeFixPreAbcdefg2241 | fliC | flagellin | 0.005 | -7.557504442 | down |
|  | GeFixPreAbcdefg1527 | - | chemotaxis protein | 0.009 | -6.768907036 | down |
|  | GeFixPreAbcdefg2979 | cheW | hypothetical protein | 0.014 | -6.188015511 | down |
|  | GeFixPreAbcdefg2148 | mcp | chemotaxis protein | 0.008 | -6.957387283 | down |
|  | GeFixPreAbcdefg2144 | - | chemotaxis protein | 0.011 | -6.510901456 | down |
|  | GeFixPreAbcdefg2145 | - | chemotaxis protein | 0.037 | -4.744679707 | down |
|  | GeFixPreAbcdefg2147 | mcp | chemotaxis protein | 0.027 | -5.191515681 | down |
|  | GeFixPreAbcdefg2140 | - | chemotaxis protein | 0.06 | -4.047962164 | down |
|  | GeFixPreAbcdefg2143 | mcp | chemotaxis protein | 0.038 | -4.732596119 | down |
|  | GeFixPreAbcdefg2254 | cheV | conserved hypothetical protein | 0.006 | -7.340500958 | down |
|  | GeFixPreAbcdefg2256 | flgM | flagellar biosynthesis anti-sigma factor FlgM | 0.01 | -6.642274405 | down |
|  | GeFixPreAbcdefg2981 | cheR | SAM-dependent methyltransferase | 0.016 | -5.979120788 | down |
|  | GeFixPreAbcdefg2982 | cheB | chemotaxis response regulator protein-glutamate methylesterase | 0.107 | -3.230279896 | down |
|  | GeFixPreAbcdefg3843 | cydA | cytochrome D ubiquinol oxidase subunit I | 0.456 | -1.133227967 | down |
|  | GeFixPreAbcdefg3844 | cydB | cytochrome d ubiquinol oxidase subunit II | 0.434 | -1.203956467 | down |
|  | GeFixPreAbcdefg3798 | motA | flagellar motor protein MotA | 0.077 | -3.702871603 | down |
|  | GeFixPreAbcdefg2263 | mcp | methyl-accepting chemotaxis (MCP) signaling domain protein | 0.008 | -6.943721866 | down |
|  | GeFixPreAbcdefg3243 | pilH | two-component system response regulator protein | 0.259 | -1.946866594 | down |
|  | GeFixPreAbcdefg2573 | mcp | chemotaxis protein | 0.011 | -6.474501389 | down |
|  | GeFixPreAbcdefg2572 | cheW | hypothetical protein | 0.013 | -6.295279905 | down |
|  | GeFixPreAbcdefg3403 | cbhA | 1,4-beta-cellobiosidase | 0.235 | -2.092195457 | down |
|  | GeFixPreAbcdefg2160 | motA | flagellar motor protein | 0.018 | -5.775680519 | down |
|  | GeFixPreAbcdefg3565 | tar | chemotaxis protein | 0.305 | -1.713181174 | down |
|  | GeFixPreAbcdefg1615 | mprF | hypothetical protein | 0.291 | -1.780990015 | down |
|  | GeFixPreAbcdefg2116 | rpfG | two-component system response regulator | 0.037 | -4.768312273 | down |
|  | GeFixPreAbcdefg2118 | rpfC | hybrid sensor histidine kinase/response regulator | 0.112 | -3.160223396 | down |
|  | GeFixPreAbcdefg2192 | fliA | RNA polymerase sigma factor FliA | 0.002 | -8.741610827 | down |
|  | GeFixPreAbcdefg2191 | cheY | response regulator | 0.005 | -7.574619209 | down |
|  | GeFixPreAbcdefg3110 | cydB | cytochrome d ubiquinol oxidase subunit II | 2.346 | 1.230275384 | up |
|  | GeFixPreAbcdefg2189 | cheA | chemotaxis protein CheA | 0.009 | -6.818910206 | down |
|  | GeFixPreAbcdefg1677 | glnD | bifunctional uridylyltransferase/uridylyl-removing protein | 3.456 | 1.789184155 | up |
|  | GeFixPreAbcdefg2135 | cheW | chemotaxis protein CheW | 0.006 | -7.417437414 | down |
|  | GeFixPreAbcdefg2978 | mcp | chemotaxis protein | 0.008 | -6.981041362 | down |
|  | GeFixPreAbcdefg2137 | mcp | chemotaxis protein | 0.005 | -7.603015942 | down |
|  | GeFixPreAbcdefg1981 | mcp | chemotaxis protein | 0.2 | -2.319585371 | down |
|  | GeFixPreAbcdefg2133 | mcp | chemotaxis protein | 0.023 | -5.453577802 | down |
|  | GeFixPreAbcdefg2132 | cheR | chemotaxis protein CheR | 0.004 | -7.954664536 | down |
|  | GeFixPreAbcdefg2977 | cheA | chemotaxis protein CheA | 0.017 | -5.83961953 | down |
|  | GeFixPreAbcdefg0030 | - | cellulase | 0.002 | -9.228895472 | down |
|  | GeFixPreAbcdefg2434 | mcp | HAMP domain-containing protein | 0.293 | -1.770731663 | down |
|  | GeFixPreAbcdefg3354 | mcp | chemotaxis protein | 0.053 | -4.241791189 | down |
|  | GeFixPreAbcdefg3597 | dctA | C4-dicarboxylate transporter | 2.802 | 1.486476422 | up |
| Histidine metabolism | GeFixPreAbcdefg2078 | hisF | imidazole glycerol phosphate synthase subunit HisF | 2.185 | 1.127820146 | up |
|  | GeFixPreAbcdefg2079 | hisIE | bifunctional phosphoribosyl-AMP cyclohydrolase/phosphoribosyl-ATP diphosphatase | 4.28 | 2.09773945 | up |
|  | GeFixPreAbcdefg2074 | hisC | histidinol-phosphate transaminase | 2.538 | 1.343586473 | up |
|  | GeFixPreAbcdefg2075 | hisB | bifunctional imidazole glycerol-phosphate dehydratase/histidinol phosphatase | 2.664 | 1.413657472 | up |
|  | GeFixPreAbcdefg2076 | hisH | imidazole glycerol phosphate synthase, glutamine amidotransferase subunit | 3.003 | 1.586320416 | up |
|  | GeFixPreAbcdefg2077 | hisA | 1-(5-phosphoribosyl)-5-[(5-phosphoribosylamino)methylideneamino]imidazole-4-carboxamide isomerase | 2.621 | 1.389874614 | up |
|  | GeFixPreAbcdefg1880 | hutH | histidine ammonia-lyase | 0.248 | -2.013836431 | down |
|  | GeFixPreAbcdefg1881 | hutI | imidazolonepropionase | 0.389 | -1.360876575 | down |
|  | GeFixPreAbcdefg1882 | - | formimidoylglutamate deiminase | 0.361 | -1.469592703 | down |
|  | GeFixPreAbcdefg1879 | hutG | N-formylglutamate deformylase | 0.278 | -1.845773367 | down |
|  | GeFixPreAbcdefg1878 | hutU | urocanate hydratase | 0.193 | -2.370047999 | down |
|  | GeFixPreAbcdefg3533 | aofH | flavin monoamine oxidase | 0.034 | -4.870458168 | down |
|  | GeFixPreAbcdefg3530 | hisC | aminotransferase | 0.07 | -3.839729995 | down |
| Peptidoglycan biosynthesis | GeFixPreAbcdefg0991 | ddl | D-alanine--D-alanine ligase | 9.28 | 3.214158974 | up |
|  | GeFixPreAbcdefg0990 | murC | UDP-N-acetylmuramate--L-alanine ligase | 6.919 | 2.790528781 | up |
|  | GeFixPreAbcdefg0989 | murG | undecaprenyldiphospho-muramoylpentapeptide beta-N-acetylglucosaminyltransferase | 5.972 | 2.578113184 | up |
|  | GeFixPreAbcdefg0987 | mraY | phospho-N-acetylmuramoyl-pentapeptide-transferase | 5.765 | 2.527333998 | up |
|  | GeFixPreAbcdefg0984 | ftsI | cell division protein | 7.21 | 2.850059643 | up |
|  | GeFixPreAbcdefg0985 | murE | UDP-N-acetylmuramoyl-L-alanyl-D-glutamate--2,6-diaminopimelate ligase | 4.454 | 2.155219707 | up |
|  | GeFixPreAbcdefg0986 | murF | UDP-N-acetylmuramoylalanyl-D-glutamyl-2, 6-diaminopimelate--D-alanyl-D-alanine ligase | 3.626 | 1.858538758 | up |
|  | GeFixPreAbcdefg3489 | mrcA | peptidase | 2.577 | 1.365840257 | up |
|  | GeFixPreAbcdefg0835 | dacC | serine-type D-Ala-D-Ala carboxypeptidase | 3.032 | 1.60032059 | up |
|  | GeFixPreAbcdefg2046 | bcrC | hypothetical protein | 3.143 | 1.651912852 | up |
| Lysine biosynthesis | GeFixPreAbcdefg0985 | murE | UDP-N-acetylmuramoyl-L-alanyl-D-glutamate--2,6-diaminopimelate ligase | 4.454 | 2.155219707 | up |
|  | GeFixPreAbcdefg0986 | murF | UDP-N-acetylmuramoylalanyl-D-glutamyl-2, 6-diaminopimelate--D-alanyl-D-alanine ligase | 3.626 | 1.858538758 | up |
|  | GeFixPreAbcdefg0801 | dapF | diaminopimelate epimerase | 2.221 | 1.150916419 | up |
|  | GeFixPreAbcdefg2101 | - | 2-aminoadipate aminotransferase | 2.759 | 1.463912686 | up |
|  | GeFixPreAbcdefg1680 | dapE | succinyl-diaminopimelate desuccinylase | 2.811 | 1.491165758 | up |
|  | GeFixPreAbcdefg2064 | thrA | bifunctional aspartate kinase/homoserine dehydrogenase I | 0.146 | -2.773124145 | down |
|  | GeFixPreAbcdefg1678 | dapD | 2,3,4,5-tetrahydropyridine-2,6-dicarboxylate N-succinyltransferase | 2.882 | 1.527214541 | up |
| Terpenoid backbone biosynthesis | GeFixPreAbcdefg0121 | STE14 | protein-S-isoprenylcysteine methyltransferase | 0.377 | -1.406067692 | down |
|  | GeFixPreAbcdefg2880 | ispA | geranyl transferase | 2.105 | 1.073741383 | up |
|  | GeFixPreAbcdefg2045 | gcpE | 4-hydroxy-3-methylbut-2-en-1-yl diphosphate synthase | 3.662 | 1.872462929 | up |
|  | GeFixPreAbcdefg1955 | ispD | 2-C-methyl-D-erythritol 4-phosphate cytidylyltransferase | 2.02 | 1.014608455 | up |
|  | GeFixPreAbcdefg2710 | dxs | 1-deoxy-D-xylulose-5-phosphate synthase | 2.767 | 1.468394642 | up |
|  | GeFixPreAbcdefg1662 | dxr | 1-deoxy-D-xylulose-5-phosphate reductoisomerase | 6.066 | 2.600820368 | up |
| Phenylalanine metabolism | GeFixPreAbcdefg1523 | katG | catalase/peroxidase HPI | 0.2 | -2.319203641 | down |
|  | GeFixPreAbcdefg0523 | hppD | 4-hydroxyphenylpyruvate dioxygenase | 4.637 | 2.213310111 | up |
|  | GeFixPreAbcdefg2074 | hisC | histidinol-phosphate transaminase | 2.538 | 1.343586473 | up |
|  | GeFixPreAbcdefg2869 | dadA | amino acid dehydrogenase | 2.245 | 1.166617556 | up |
|  | GeFixPreAbcdefg3533 | aofH | flavin monoamine oxidase | 0.034 | -4.870458168 | down |
|  | GeFixPreAbcdefg3530 | hisC | aminotransferase | 0.07 | -3.839729995 | down |
| Cell cycle | GeFixPreAbcdefg0993 | ftsA | cell division protein FtsA | 5.579 | 2.480072268 | up |
|  | GeFixPreAbcdefg0992 | ftsQ | cell division protein FtsQ | 9.743 | 3.284322386 | up |
|  | GeFixPreAbcdefg0995 | ftsZ | cell division protein FtsZ | 3.843 | 1.942268281 | up |
|  | GeFixPreAbcdefg0988 | ftsW | cell division protein FtsW | 6.54 | 2.709270703 | up |
|  | GeFixPreAbcdefg0989 | murG | undecaprenyldiphospho-muramoylpentapeptide beta-N-acetylglucosaminyltransferase | 5.972 | 2.578113184 | up |
| Tyrosine metabolism | GeFixPreAbcdefg0523 | hppD | 4-hydroxyphenylpyruvate dioxygenase | 4.637 | 2.213310111 | up |
|  | GeFixPreAbcdefg2074 | hisC | histidinol-phosphate transaminase | 2.538 | 1.343586473 | up |
|  | GeFixPreAbcdefg3709 | faaH | 2-keto-4-pentenoate hydratase | 3.078 | 1.621985229 | up |
|  | GeFixPreAbcdefg3708 | maiA | maleylacetoacetate isomerase | 5.645 | 2.496917714 | up |
|  | GeFixPreAbcdefg3533 | aofH | flavin monoamine oxidase | 0.034 | -4.870458168 | down |
|  | GeFixPreAbcdefg3530 | hisC | aminotransferase | 0.07 | -3.839729995 | down |
| Vancomycin resistance | GeFixPreAbcdefg0991 | ddl | D-alanine--D-alanine ligase | 9.28 | 3.214158974 | up |
|  | GeFixPreAbcdefg0989 | murG | undecaprenyldiphospho-muramoylpentapeptide beta-N-acetylglucosaminyltransferase | 5.972 | 2.578113184 | up |
|  | GeFixPreAbcdefg0987 | mraY | phospho-N-acetylmuramoyl-pentapeptide-transferase | 5.765 | 2.527333998 | up |
|  | GeFixPreAbcdefg0986 | murF | UDP-N-acetylmuramoylalanyl-D-glutamyl-2, 6-diaminopimelate--D-alanyl-D-alanine ligase | 3.626 | 1.858538758 | up |

**Table S6 The expression of DEGs in KEGG enrichment pathways of 6d_GC2 vs GC1**

| **Pathway** | **Gene_id** | **Gene name** | **Gene description** | **FC(GC2_6/GC1_6)** | **Log2FC(GC2_6/GC1_6)** | **Regulate** |
| --- | --- | --- | --- | --- | --- | --- |
| Bacterial chemotaxis | GeFixPreAbcdefg2159 | motB | flagellar motor protein MotD | 0.137 | -2.865 | down |
|  | GeFixPreAbcdefg2157 | cheW | chemotaxis protein | 0.166 | -2.59417 | down |
|  | GeFixPreAbcdefg2155 | cheY | Fis family transcriptional regulator | 0.244 | -2.03505 | down |
|  | GeFixPreAbcdefg2154 | cheA | chemotaxis protein CheA | 0.181 | -2.4684 | down |
|  | GeFixPreAbcdefg2153 | - | chemotaxis protein | 0.192 | -2.38168 | down |
|  | GeFixPreAbcdefg2151 | - | chemotaxis protein | 0.174 | -2.5205 | down |
|  | GeFixPreAbcdefg1527 | - | chemotaxis protein | 0.088 | -3.50129 | down |
|  | GeFixPreAbcdefg2979 | cheW | hypothetical protein | 0.123 | -3.01917 | down |
|  | GeFixPreAbcdefg2148 | mcp | chemotaxis protein | 0.19 | -2.39823 | down |
|  | GeFixPreAbcdefg2144 | - | chemotaxis protein | 0.22 | -2.18594 | down |
|  | GeFixPreAbcdefg2145 | - | chemotaxis protein | 0.258 | -1.95623 | down |
|  | GeFixPreAbcdefg2147 | mcp | chemotaxis protein | 0.229 | -2.12603 | down |
|  | GeFixPreAbcdefg2140 | - | chemotaxis protein | 0.334 | -1.58246 | down |
|  | GeFixPreAbcdefg2143 | mcp | chemotaxis protein | 0.2 | -2.32021 | down |
|  | GeFixPreAbcdefg2254 | cheV | conserved hypothetical protein | 0.154 | -2.70251 | down |
|  | GeFixPreAbcdefg2981 | cheR | SAM-dependent methyltransferase | 0.157 | -2.66899 | down |
|  | GeFixPreAbcdefg2982 | cheB | chemotaxis response regulator protein-glutamate methylesterase | 0.195 | -2.35861 | down |
|  | GeFixPreAbcdefg3798 | motA | flagellar motor protein MotA | 0.251 | -1.99382 | down |
|  | GeFixPreAbcdefg3799 | motB | flagellar motor protein MotB | 0.214 | -2.22546 | down |
|  | GeFixPreAbcdefg2263 | mcp | methyl-accepting chemotaxis (MCP) signaling domain protein | 0.199 | -2.3323 | down |
|  | GeFixPreAbcdefg2573 | mcp | chemotaxis protein | 0.199 | -2.32825 | down |
|  | GeFixPreAbcdefg2572 | cheW | hypothetical protein | 0.218 | -2.19928 | down |
|  | GeFixPreAbcdefg2160 | motA | flagellar motor protein | 0.193 | -2.37252 | down |
|  | GeFixPreAbcdefg3565 | tar | chemotaxis protein | 0.383 | -1.38373 | down |
|  | GeFixPreAbcdefg2191 | cheY | response regulator | 0.274 | -1.86947 | down |
|  | GeFixPreAbcdefg2190 | cheZ | chemotaxis protein | 0.288 | -1.79563 | down |
|  | GeFixPreAbcdefg2219 | fliG | flagellar motor switch protein FliG | 0.147 | -2.76867 | down |
|  | GeFixPreAbcdefg2211 | fliNY | flagellar motor switch protein FliN | 0.211 | -2.24144 | down |
|  | GeFixPreAbcdefg2212 | fliM | flagellar motor switch protein FliM | 0.152 | -2.72213 | down |
|  | GeFixPreAbcdefg2189 | cheA | chemotaxis protein CheA | 0.272 | -1.87866 | down |
|  | GeFixPreAbcdefg2135 | cheW | chemotaxis protein CheW | 0.165 | -2.59571 | down |
|  | GeFixPreAbcdefg2978 | mcp | chemotaxis protein | 0.107 | -3.21834 | down |
|  | GeFixPreAbcdefg2137 | mcp | chemotaxis protein | 0.139 | -2.84768 | down |
|  | GeFixPreAbcdefg1981 | mcp | chemotaxis protein | 0.264 | -1.9212 | down |
|  | GeFixPreAbcdefg2132 | cheR | chemotaxis protein CheR | 0.234 | -2.09508 | down |
|  | GeFixPreAbcdefg2977 | cheA | chemotaxis protein CheA | 0.079 | -3.658 | down |
|  | GeFixPreAbcdefg3354 | mcp | chemotaxis protein | 0.179 | -2.47828 | down |
| Flagellar assembly | GeFixPreAbcdefg2239 | fliS | flagellar protein FliS | 0.17 | -2.55654369 | down |
|  | GeFixPreAbcdefg2159 | motB | flagellar motor protein MotD | 0.137 | -2.864995595 | down |
|  | GeFixPreAbcdefg2247 | flgG | flagellar basal body rod protein FlgG | 0.11 | -3.178275451 | down |
|  | GeFixPreAbcdefg2246 | flgH | flagellar basal body L-ring protein | 0.095 | -3.393133487 | down |
|  | GeFixPreAbcdefg2245 | flgI | flagellar P-ring protein | 0.128 | -2.969954505 | down |
|  | GeFixPreAbcdefg2243 | flgK | flagellar hook protein FlgK | 0.148 | -2.755844413 | down |
|  | GeFixPreAbcdefg2242 | flgL | flagellar hook protein FlgL | 0.198 | -2.335369981 | down |
|  | GeFixPreAbcdefg2241 | fliC | flagellin | 0.19 | -2.394975301 | down |
|  | GeFixPreAbcdefg2240 | fliD | flagellar protein | 0.205 | -2.287985719 | down |
|  | GeFixPreAbcdefg2249 | flgF | flagellar basal body rod protein FlgF | 0.156 | -2.678247955 | down |
|  | GeFixPreAbcdefg2255 | flgA | flagellar basal body P-ring biosynthesis protein FlgA | 0.169 | -2.563545201 | down |
|  | GeFixPreAbcdefg2256 | flgM | flagellar biosynthesis anti-sigma factor FlgM | 0.297 | -1.74992694 | down |
|  | GeFixPreAbcdefg2250 | flgE | flagellar hook protein FlgE | 0.161 | -2.636814513 | down |
|  | GeFixPreAbcdefg2251 | flgD | flagellar basal body rod modification protein | 0.149 | -2.748084817 | down |
|  | GeFixPreAbcdefg2252 | flgC | flagellar basal body rod protein FlgC | 0.174 | -2.525638055 | down |
|  | GeFixPreAbcdefg2253 | flgB | flagellar biosynthesis protein FlgB | 0.201 | -2.315426533 | down |
|  | GeFixPreAbcdefg3798 | motA | flagellar motor protein MotA | 0.251 | -1.993820882 | down |
|  | GeFixPreAbcdefg3799 | motB | flagellar motor protein MotB | 0.214 | -2.225464843 | down |
|  | GeFixPreAbcdefg2160 | motA | flagellar motor protein | 0.193 | -2.372519812 | down |
|  | GeFixPreAbcdefg2209 | fliP | flagellar biosynthetic protein FliP | 0.178 | -2.487175296 | down |
|  | GeFixPreAbcdefg2208 | fliQ | flagellar biosynthesis | 0.227 | -2.141655101 | down |
|  | GeFixPreAbcdefg2202 | flhB | flagellar biosynthesis protein FlhB | 0.183 | -2.446514444 | down |
|  | GeFixPreAbcdefg2201 | flhA | flagellar biosynthesis protein FlhA | 0.128 | -2.968498791 | down |
|  | GeFixPreAbcdefg2192 | fliA | RNA polymerase sigma factor FliA | 0.187 | -2.419028663 | down |
|  | GeFixPreAbcdefg2218 | fliH | flagellar assembly protein FliH | 0.24 | -2.058552057 | down |
|  | GeFixPreAbcdefg2219 | fliG | flagellar motor switch protein FliG | 0.147 | -2.768667238 | down |
|  | GeFixPreAbcdefg2210 | fliOZ | flagellar biosynthetic protein FliO | 0.172 | -2.538512978 | down |
|  | GeFixPreAbcdefg2211 | fliNY | flagellar motor switch protein FliN | 0.211 | -2.241436408 | down |
|  | GeFixPreAbcdefg2212 | fliM | flagellar motor switch protein FliM | 0.152 | -2.722128449 | down |
|  | GeFixPreAbcdefg2215 | fliK | flagellar protein | 0.286 | -1.804630766 | down |
|  | GeFixPreAbcdefg2216 | fliJ | flagellar export protein FliJ | 0.358 | -1.481113707 | down |
|  | GeFixPreAbcdefg2217 | fliI | flagellar protein export ATPase FliI | 0.206 | -2.282381903 | down |
|  | GeFixPreAbcdefg2207 | fliR | flagellar biosynthetic protein FliR | 0.197 | -2.341287834 | down |
|  | GeFixPreAbcdefg2221 | fliE | flagellar hook-basal body protein | 0.268 | -1.898048429 | down |
|  | GeFixPreAbcdefg2220 | fliF | flagellar M-ring protein FliF | 0.165 | -2.59690135 | down |
| Two-component system | GeFixPreAbcdefg2489 | - | putative membrane protein | 14.645 | 3.87229008 | up |
|  | GeFixPreAbcdefg2488 | cydB | cytochrome d ubiquinol oxidase subunit II | 7.778 | 2.959441455 | up |
|  | GeFixPreAbcdefg2235 | rpoN | RNA polymerase sigma-54 factor | 0.471 | -1.086591053 | down |
|  | GeFixPreAbcdefg1255 | phoR | histidine kinase | 0.464 | -1.108856234 | down |
|  | GeFixPreAbcdefg2157 | cheW | chemotaxis protein | 0.166 | -2.594174607 | down |
|  | GeFixPreAbcdefg2155 | cheY | Fis family transcriptional regulator | 0.244 | -2.035050958 | down |
|  | GeFixPreAbcdefg2154 | cheA | chemotaxis protein CheA | 0.181 | -2.468400428 | down |
|  | GeFixPreAbcdefg2153 | - | chemotaxis protein | 0.192 | -2.381679624 | down |
|  | GeFixPreAbcdefg2151 | - | chemotaxis protein | 0.174 | -2.520499733 | down |
|  | GeFixPreAbcdefg0031 | - | cellulase | 0.407 | -1.298298517 | down |
|  | GeFixPreAbcdefg2241 | fliC | flagellin | 0.19 | -2.394975301 | down |
|  | GeFixPreAbcdefg1527 | - | chemotaxis protein | 0.088 | -3.501287905 | down |
|  | GeFixPreAbcdefg1919 | - | cytochrome c | 3.102 | 1.633217415 | up |
|  | GeFixPreAbcdefg2979 | cheW | hypothetical protein | 0.123 | -3.019173343 | down |
|  | GeFixPreAbcdefg2148 | mcp | chemotaxis protein | 0.19 | -2.398234823 | down |
|  | GeFixPreAbcdefg2144 | - | chemotaxis protein | 0.22 | -2.185941545 | down |
|  | GeFixPreAbcdefg2145 | - | chemotaxis protein | 0.258 | -1.956226063 | down |
|  | GeFixPreAbcdefg2147 | mcp | chemotaxis protein | 0.229 | -2.12602898 | down |
|  | GeFixPreAbcdefg2140 | - | chemotaxis protein | 0.334 | -1.582457177 | down |
|  | GeFixPreAbcdefg2143 | mcp | chemotaxis protein | 0.2 | -2.320210646 | down |
|  | GeFixPreAbcdefg2254 | cheV | conserved hypothetical protein | 0.154 | -2.702509238 | down |
|  | GeFixPreAbcdefg2256 | flgM | flagellar biosynthesis anti-sigma factor FlgM | 0.297 | -1.74992694 | down |
|  | GeFixPreAbcdefg2981 | cheR | SAM-dependent methyltransferase | 0.157 | -2.668986172 | down |
|  | GeFixPreAbcdefg2982 | cheB | chemotaxis response regulator protein-glutamate methylesterase | 0.195 | -2.358610621 | down |
|  | GeFixPreAbcdefg3798 | motA | flagellar motor protein MotA | 0.251 | -1.993820882 | down |
|  | GeFixPreAbcdefg2263 | mcp | methyl-accepting chemotaxis (MCP) signaling domain protein | 0.199 | -2.332295289 | down |
|  | GeFixPreAbcdefg2573 | mcp | chemotaxis protein | 0.199 | -2.328250529 | down |
|  | GeFixPreAbcdefg2572 | cheW | hypothetical protein | 0.218 | -2.199276875 | down |
|  | GeFixPreAbcdefg2160 | motA | flagellar motor protein | 0.193 | -2.372519812 | down |
|  | GeFixPreAbcdefg3565 | tar | chemotaxis protein | 0.383 | -1.383733004 | down |
|  | GeFixPreAbcdefg1615 | mprF | hypothetical protein | 0.218 | -2.19523998 | down |
|  | GeFixPreAbcdefg2116 | rpfG | two-component system response regulator | 0.135 | -2.893688266 | down |
|  | GeFixPreAbcdefg2118 | rpfC | hybrid sensor histidine kinase/response regulator | 0.383 | -1.384798689 | down |
|  | GeFixPreAbcdefg2043 | regA | chemotaxis protein CheY | 2.626 | 1.393138996 | up |
|  | GeFixPreAbcdefg2192 | fliA | RNA polymerase sigma factor FliA | 0.187 | -2.419028663 | down |
|  | GeFixPreAbcdefg2191 | cheY | response regulator | 0.274 | -1.869470457 | down |
|  | GeFixPreAbcdefg1818 | pstS | phosphate ABC transporter substrate-binding protein PstS | 0.444 | -1.170901886 | down |
|  | GeFixPreAbcdefg2189 | cheA | chemotaxis protein CheA | 0.272 | -1.878656474 | down |
|  | GeFixPreAbcdefg2135 | cheW | chemotaxis protein CheW | 0.165 | -2.595708946 | down |
|  | GeFixPreAbcdefg2978 | mcp | chemotaxis protein | 0.107 | -3.218335473 | down |
|  | GeFixPreAbcdefg2137 | mcp | chemotaxis protein | 0.139 | -2.847675546 | down |
|  | GeFixPreAbcdefg1981 | mcp | chemotaxis protein | 0.264 | -1.921202428 | down |
|  | GeFixPreAbcdefg2132 | cheR | chemotaxis protein CheR | 0.234 | -2.095079479 | down |
|  | GeFixPreAbcdefg2977 | cheA | chemotaxis protein CheA | 0.079 | -3.65799555 | down |
|  | GeFixPreAbcdefg0030 | - | cellulase | 0.043 | -4.531208627 | down |
|  | GeFixPreAbcdefg3193 | ampC | serine hydrolase | 0.401 | -1.31681 | down |
|  | GeFixPreAbcdefg3354 | mcp | chemotaxis protein | 0.179 | -2.47828 | down |
|  | GeFixPreAbcdefg3597 | dctA | C4-dicarboxylate transporter | 0.165 | -2.603 | down |
| Histidine metabolism | GeFixPreAbcdefg1882 | - | formimidoylglutamate deiminase | 0.221 | -2.178526884 | down |
|  | GeFixPreAbcdefg2072 | hisG | ATP phosphoribosyltransferase | 0.159 | -2.650028075 | down |
|  | GeFixPreAbcdefg2073 | hisD | histidinol dehydrogenase | 0.161 | -2.633596898 | down |
|  | GeFixPreAbcdefg2074 | hisC | histidinol-phosphate transaminase | 0.267 | -1.907651668 | down |
|  | GeFixPreAbcdefg2075 | hisB | bifunctional imidazole glycerol-phosphate dehydratase/histidinol phosphatase | 0.437 | -1.193279127 | down |
|  | GeFixPreAbcdefg2076 | hisH | imidazole glycerol phosphate synthase, glutamine amidotransferase subunit | 0.48 | -1.059889757 | down |
|  | GeFixPreAbcdefg3533 | aofH | flavin monoamine oxidase | 6.095 | 2.607589066 | up |
|  | GeFixPreAbcdefg1880 | hutH | histidine ammonia-lyase | 0.265 | -1.918006647 | down |
|  | GeFixPreAbcdefg1881 | hutI | imidazolonepropionase | 0.266 | -1.908365387 | down |
|  | GeFixPreAbcdefg1879 | hutG | N-formylglutamate deformylase | 0.262 | -1.93200865 | down |
|  | GeFixPreAbcdefg1878 | hutU | urocanate hydratase | 0.249 | -2.00476604 | down |
|  | GeFixPreAbcdefg3530 | hisC | aminotransferase | 5.275 | 2.399121243 | up |
| Tyrosine metabolism | GeFixPreAbcdefg0523 | hppD | 4-hydroxyphenylpyruvate dioxygenase | 7.037 | 2.81498827 | up |
|  | GeFixPreAbcdefg0524 | hmgA | homogentisate 1,2-dioxygenase | 2.479 | 1.30979718 | up |
|  | GeFixPreAbcdefg0110 | adhP | zinc-dependent alcohol dehydrogenase | 3.568 | 1.835188164 | up |
|  | GeFixPreAbcdefg2074 | hisC | histidinol-phosphate transaminase | 0.267 | -1.907651668 | down |
|  | GeFixPreAbcdefg3533 | aofH | flavin monoamine oxidase | 6.095 | 2.607589066 | up |
|  | GeFixPreAbcdefg3709 | faaH | 2-keto-4-pentenoate hydratase | 4.252 | 2.087994647 | up |
|  | GeFixPreAbcdefg3708 | maiA | maleylacetoacetate isomerase | 3.255 | 1.702690497 | up |
|  | GeFixPreAbcdefg3530 | hisC | aminotransferase | 5.275 | 2.399121243 | up |
| Sulfur metabolism | GeFixPreAbcdefg3176 | metX | homoserine acetyltransferase | 0.268 | -1.897753215 | down |
|  | GeFixPreAbcdefg3417 | cysNC | adenylyl-sulfate kinase | 0.401 | -1.319217591 | down |
|  | GeFixPreAbcdefg3075 | cysJ | sulfite reductase | 0.432 | -1.211130032 | down |
|  | GeFixPreAbcdefg3420 | cysI | sulfite reductase subunit beta | 0.329 | -1.602286198 | down |
|  | GeFixPreAbcdefg3421 | cysH | phosphoadenosine phosphosulfate reductase | 0.417 | -1.262511398 | down |
|  | GeFixPreAbcdefg1062 | ssuB | sulfonate ABC transporter ATP-binding protein | 0.117 | -3.091390323 | down |
|  | GeFixPreAbcdefg1063 | ssuC | ABC transporter permease | 0.134 | -2.901988776 | down |
|  | GeFixPreAbcdefg1066 | ssuE | FMN reductase | 0.204 | -2.289977333 | down |
|  | GeFixPreAbcdefg1064 | ssuA | ABC transporter substrate-binding protein | 0.234 | -2.092921891 | down |
|  | GeFixPreAbcdefg1065 | ssuD | alkanesulfonate monooxygenase | 0.233 | -2.102161817 | down |
|  | GeFixPreAbcdefg0407 | ssuE | FMN reductase | 9.589 | 3.261441053 | up |
| C5-Branched dibasic acid metabolism | GeFixPreAbcdefg3575 | leuB | 3-isopropylmalate dehydrogenase | 2.382 | 1.251871639 | up |
|  | GeFixPreAbcdefg3576 | leuD | 3-isopropylmalate dehydratase small subunit | 3.166 | 1.662676973 | up |
|  | GeFixPreAbcdefg3570 | ilvB | acetolactate synthase 2 catalytic subunit | 0.251 | -1.994089217 | down |
|  | GeFixPreAbcdefg3577 | leuC | 3-isopropylmalate dehydratase large subunit | 3.791 | 1.922753294 | up |
| Valine, leucine and isoleucine biosynthesis | GeFixPreAbcdefg3575 | leuB | 3-isopropylmalate dehydrogenase | 2.382 | 1.251871639 | up |
|  | GeFixPreAbcdefg3569 | ilvC | ketol-acid reductoisomerase | 0.179 | -2.481592586 | down |
|  | GeFixPreAbcdefg3576 | leuD | 3-isopropylmalate dehydratase small subunit | 3.166 | 1.662676973 | up |
|  | GeFixPreAbcdefg3570 | ilvB | acetolactate synthase 2 catalytic subunit | 0.251 | -1.994089217 | down |
|  | GeFixPreAbcdefg3577 | leuC | 3-isopropylmalate dehydratase large subunit | 3.791 | 1.922753294 | up |
| Phenylalanine metabolism | GeFixPreAbcdefg1523 | katG | catalase/peroxidase HPI | 0.37 | -1.436065817 | down |
|  | GeFixPreAbcdefg0523 | hppD | 4-hydroxyphenylpyruvate dioxygenase | 7.037 | 2.81498827 | up |
|  | GeFixPreAbcdefg2074 | hisC | histidinol-phosphate transaminase | 0.267 | -1.907651668 | down |
|  | GeFixPreAbcdefg3533 | aofH | flavin monoamine oxidase | 6.095 | 2.607589066 | up |
|  | GeFixPreAbcdefg3530 | hisC | aminotransferase | 5.275 | 2.399121243 | up |
| Glucagon signaling | GeFixPreAbcdefg0515 | pdhB | 2-oxoisovalerate dehydrogenase | 6.495 | 2.699427221 | up |
|  | GeFixPreAbcdefg0516 | pdhA | pyruvate dehydrogenase (acetyl-transferring) E1 component subunit alpha | 7.314 | 2.870629621 | up |
|  | GeFixPreAbcdefg1008 | gpmA | hypothetical protein | 10.405 | 3.379207212 | up |
| Biofilm formation | GeFixPreAbcdefg1116 | oxyR | DNA-binding transcriptional regulator OxyR | 0.148 | -2.751804309 | down |
|  | GeFixPreAbcdefg2256 | flgM | flagellar biosynthesis anti-sigma factor FlgM | 0.297 | -1.74992694 | down |
|  | GeFixPreAbcdefg2192 | fliA | RNA polymerase sigma factor FliA | 0.187 | -2.419028663 | down |
|  | GeFixPreAbcdefg1766 | oxyR | hyaluronan synthase | 0.332 | -1.59211699 | down |
|  | GeFixPreAbcdefg0931 | bcsA | cellulose synthase catalytic subunit (UDP-forming) | 0.289 | -1.788995654 | down |
| Central carbon metabolism | GeFixPreAbcdefg0515 | pdhB | 2-oxoisovalerate dehydrogenase | 6.495 | 2.699427221 | up |
|  | GeFixPreAbcdefg0516 | pdhA | pyruvate dehydrogenase (acetyl-transferring) E1 component subunit alpha | 7.314 | 2.870629621 | up |
|  | GeFixPreAbcdefg1008 | gpmA | hypothetical protein | 10.405 | 3.379207212 | up |
| Styrene degradation | GeFixPreAbcdefg0524 | hmgA | homogentisate 1,2-dioxygenase | 2.479 | 1.30979718 | up |
|  | GeFixPreAbcdefg3709 | faaH | 2-keto-4-pentenoate hydratase | 4.252 | 2.087994647 | up |
|  | GeFixPreAbcdefg3708 | maiA | maleylacetoacetate isomerase | 3.255 | 1.702690497 | up |
| Glycolysis | GeFixPreAbcdefg0110 | adhP | zinc-dependent alcohol dehydrogenase | 3.568 | 1.835188164 | up |
|  | GeFixPreAbcdefg0515 | pdhB | 2-oxoisovalerate dehydrogenase | 6.495 | 2.699427221 | up |
|  | GeFixPreAbcdefg0516 | pdhA | pyruvate dehydrogenase (acetyl-transferring) E1 component subunit alpha | 7.314 | 2.870629621 | up |
|  | GeFixPreAbcdefg0511 | aceF | branched-chain alpha-keto acid dehydrogenase subunit E2 | 2.044 | 1.031621818 | up |
|  | GeFixPreAbcdefg0513 | aceF | branched-chain alpha-keto acid dehydrogenase subunit E2 | 2.713 | 1.440044977 | up |
|  | GeFixPreAbcdefg0109 | aldB | aldehyde dehydrogenase | 6.363 | 2.669765507 | up |
|  | GeFixPreAbcdefg2818 | tpiA | triose-phosphate isomerase | 2.437 | 1.284877012 | up |
|  | GeFixPreAbcdefg1008 | gpmA | hypothetical protein | 10.405 | 3.379207212 | up |
